# Supplementary material for: A structure-based designed small molecule depletes hRpn13Pru and a select group of KEN box proteins
Source: Nat Commun. 2024 Mar 20;15:2485. doi: 10.1038/s41467-024-46644-7 (PMC10954691; doi:10.1038/s41467-024-46644-7)

Supplementary Information

A structure-based designed small molecule depletes hRpn13^{Pru} and a select group of KEN box proteins

Xiuxiu Lu[1], Monika Chandravanshi[1], Venkata R. Sabbasani[2], Snehal Gaikwad[3], V. Keith Hughitt[3], Nana Gyabaah-Kessie[3], Bradley T Scroggins[4], Sudipto Das[5], Wazo Myint[6], Michelle E. Clapp[7], Charles D. Schwieters[8], Marzena A. Dyba[9], Derek L. Bolhuis[10], Janusz W. Koscielniak[11], Thorkell Andresson[5], Michael J. Emanuele[12], Nicholas G. Brown[12], Hiroshi Matsuo[6], Raj Chari[7], Deborah E. Citrin[4], Beverly A. Mock[3], Rolf E. Swenson[2], Kylie J. Walters[1,*]

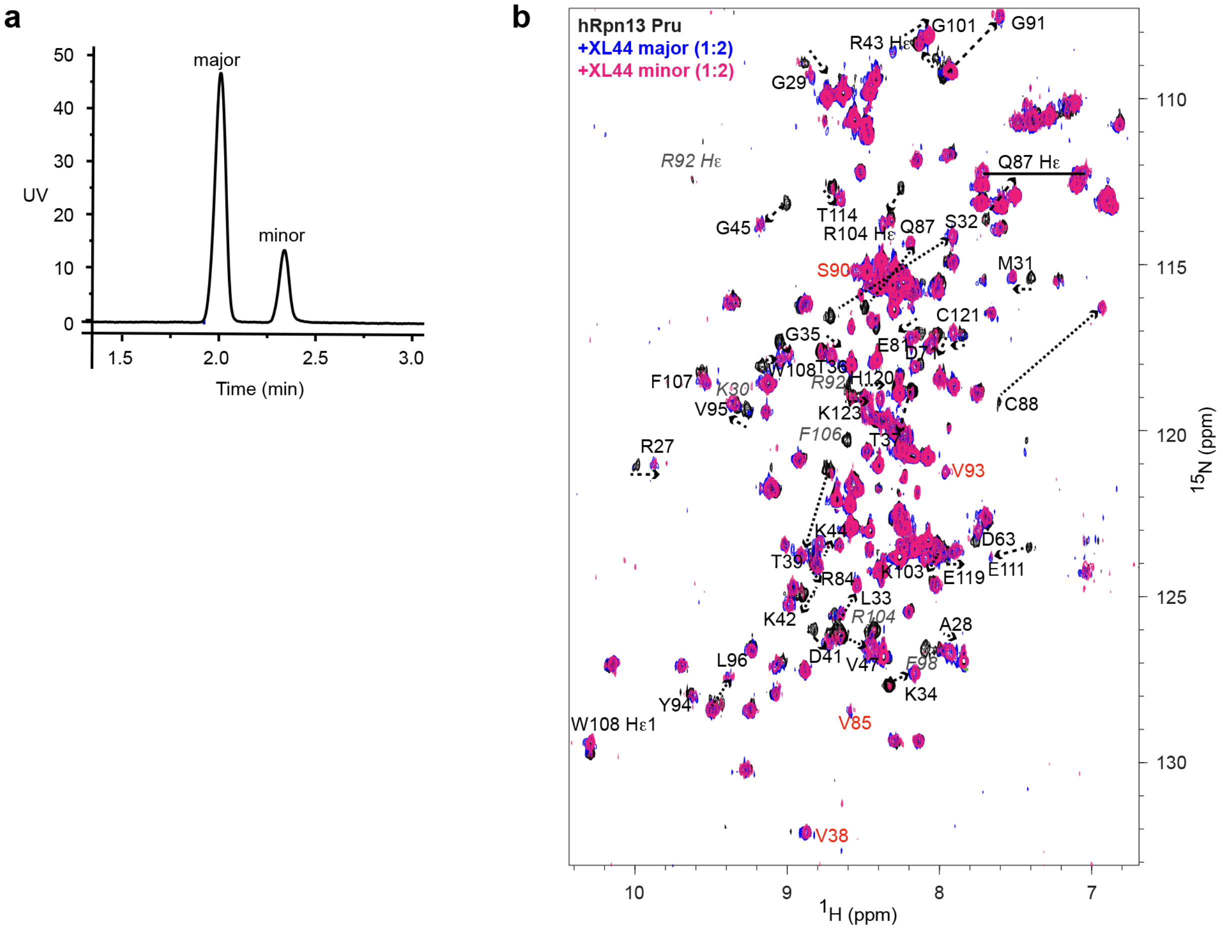

Supplementary Fig. 1 Biophysical analyses of hRpn13 binding to XL44 stereoisomers. **a**, LC-MS UV chromatogram of commercial **XL44**. **b**, ^{1}H, ^{15}N HSQC spectra of 20 μM ^{15}N-labeled hRpn13 Pru with DMSO (vehicle control, black) or addition of 2-fold molar excess **XL44** major (blue) or minor (pink) stereoisomer. The definition of major versus minor stereoisomer is based on panel a. Spectra were acquired at 600 MHz and 10°C.

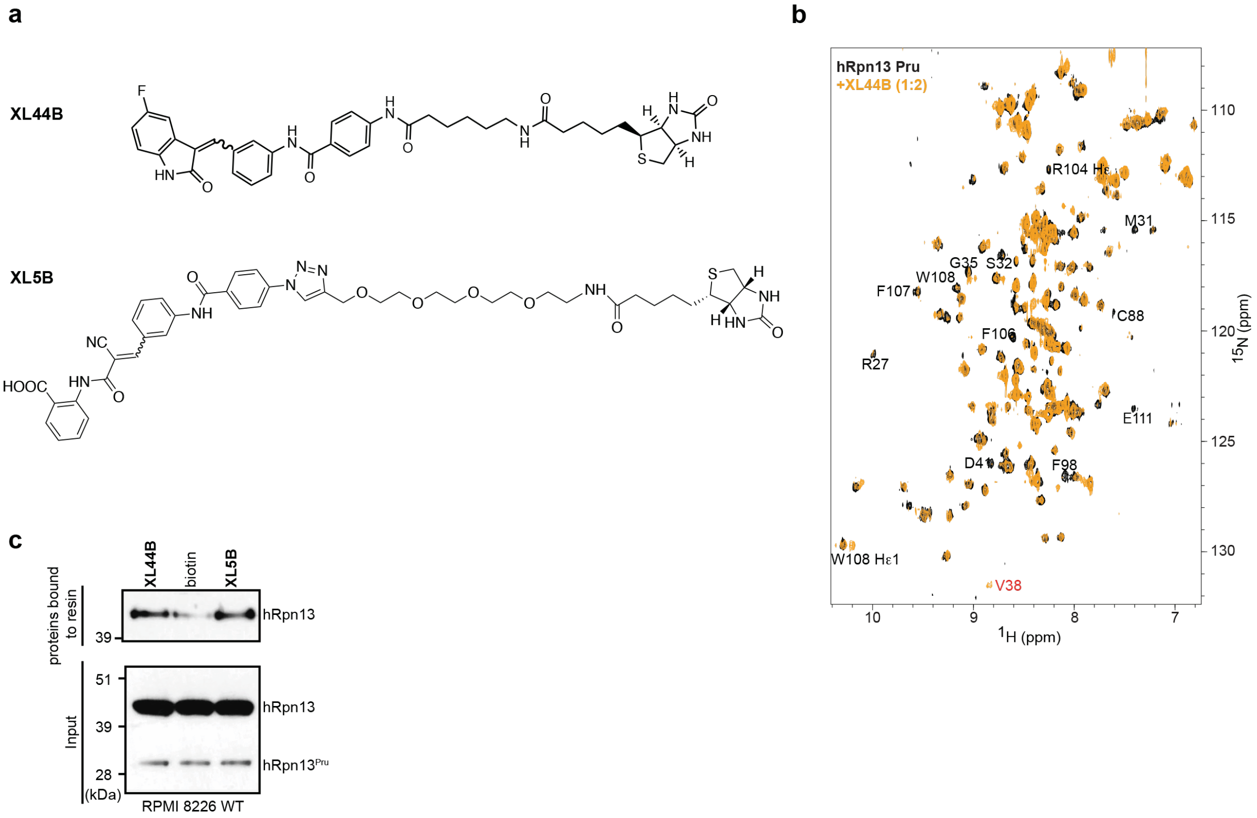

Supplementary Fig. 2 Biotinylated XL44 isolates hRpn13 from RPMI 8226 lysates. **a**, Chemical structures of biotinylated **XL44** (**XL44B**) and **XL5** (**XL5B**). **b,** ^{1}H, ^{15}N HSQC spectra of 20 µM ^{15}N-labeled hRpn13 Pru with DMSO (vehicle control, black) or 2-fold molar excess **XL44B**. Spectra were acquired at 600 MHz and 10°C. **c**, Immunoblots with antibodies against hRpn13 of RPMI 8226 lysates following incubation with 40 µM **XL5B**, biotin (negative control) or **XL44B** (bottom panel) and corresponding biotin pulldowns with streptavidin (top panel). For the pulldown experiments, the lysates were incubated with streptavidin and unbound proteins removed by washing.

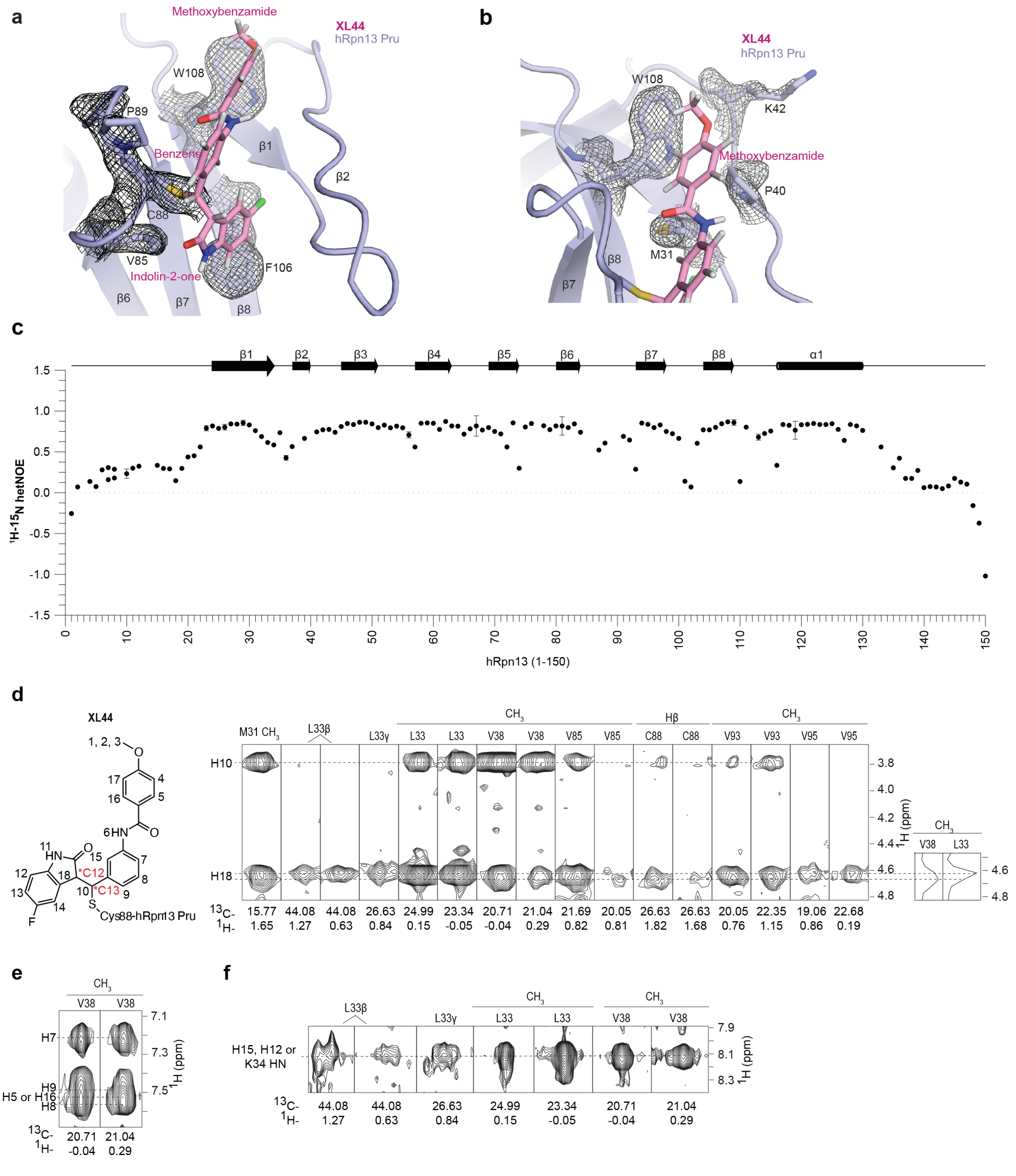

Supplementary Fig. 3 X-ray density map and NMR data for XL44-bound hRpn13 Pru. **a-b**, Electron density map for the hRpn13 sidechain atoms of Val85, Cys88, Pro89, Phe106 and Trp108 (**a**) or Met31, Pro40, Lys42, and Trp108 (**b**) in the **XL44**-ligated crystal structure with the surrounding hRpn13 ribbon structure (purple) and XL44 region

displayed. Black mesh indicates the 2$Fo - Fc$ electron density map at 1.7σ for the hRpn13 sidechain residues shown with purple stick rendering. **XL44** is displayed in stick rendering with carbon, oxygen, nitrogen, and hydrogen in pink, red, indigo, and grey, respectively. **c,** ^{1}H-^{15}N heteronuclear NOE experiments were acquired on 0.44 mM hRpn13 Pru at 25 °C and 850 MHz. Prolines and unassigned residues (Val38, Val85 and Ser90) are not plotted. Heteronuclear NOE error was estimated from spectrum noise and secondary structural elements are defined above the plot. **d-f**, Chemical structure of **XL44** including the ligated sulfur atom from hRpn13 Cys88. Hydrogen atoms are labeled with the numbers used in the text and figures. Chiral centers C12 and C13 are labeled and indicated with stars in red font (**d**, left panel). Selected regions focusing on **XL44** H10 and H18 (**d**, right panel), the **XL44** methyl group (**d**), or hRpn13 Leu33 and Val38 (**e**-**f**) from a ^{1}H, ^{13}C half-filtered NOESY (100 ms) experiment (right panel) acquired on a sample containing 0.4 mM ^{13}C-labeled hRpn13 Pru and equimolar unlabeled **XL44** dissolved in NMR buffer. In (**d**), a 1D ^{1}H trace is displayed to the right for Leu33 and Val38 methyl groups indicating the presence of two chemical shift values for **XL44** proton H18.

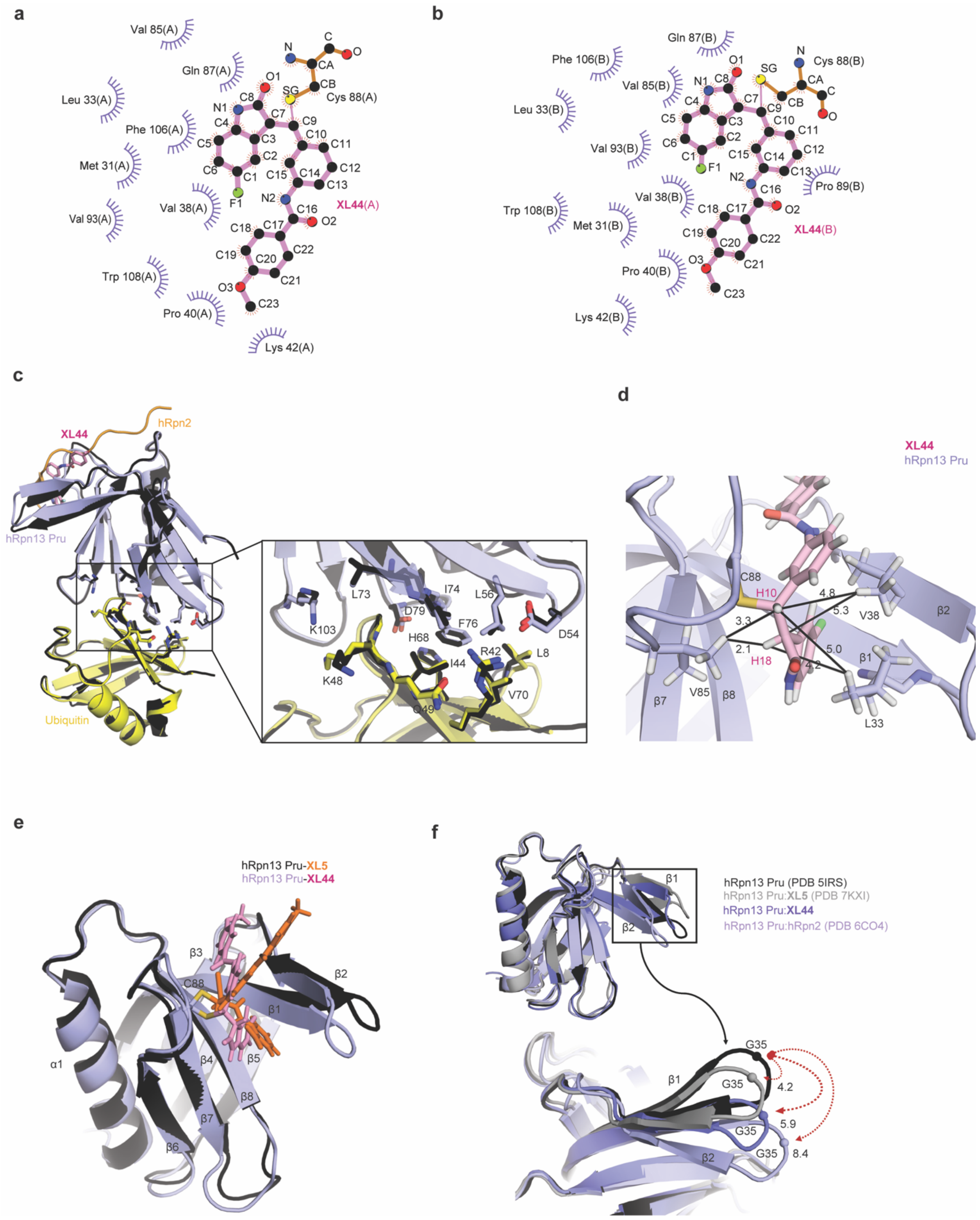

Supplementary Fig. 4 Comparative structural analyses of hRpn13 Pru alone or with XL5, XL44, or hRpn2 (940-953). a, **b**, Interaction network for **XL44** when bound to the

A- (**a**) or B- (**b**) chain of hRpn13 Pru. The definition of these two chains for the two molecules present in the asymmetric unit is arbitrary. Ligplot analyses shows similar hydrophobic interactions between **XL44** and hRpn13 Pru from either chain. **c,** Structural superimposition of the hRpn13 Pru:ubiquitin:**XL44** ternary complex with hRpn13 Pru:ubiquitin:hRpn2 (black, PDB 5V1Y) depicts an identical interface between hRpn13 (purple) and ubiquitin (yellow). Interface residues are represented with stick rendering. **d,** Model of **XL44**-ligated hRpn13 Pru with S, S stereochemistry for the two chiral centers C12 and C13. **e**, Overlay of hRpn13 Pru structure bound with **XL5** (orange, PDB 7KXI) or **XL44** (pink). **f,** Structural comparison of apo and bound-state structures of hRpn13 Pru by aligning backbone atom Cα. Changes in the β1 - β2 hairpin upon binding to different molecules is highlighted by red dotted arrows and distances are shown for the Gly35 Cα atom relative to the apo structure.

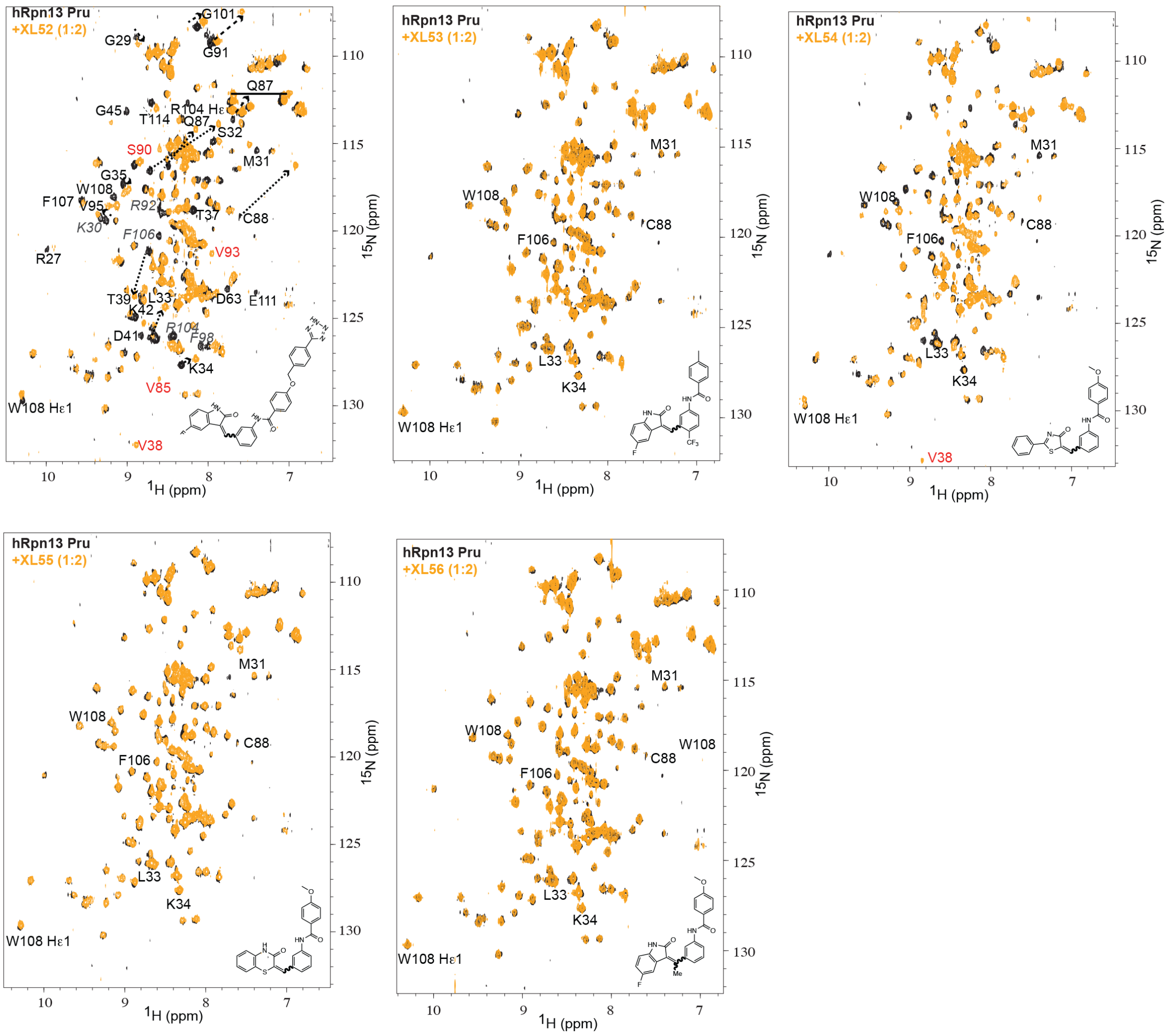

Supplementary Fig. 5 2D NMR analyses of XL44 derivatives. ^{1}H, ^{15}N HSQC spectra of 20 μM ^{15}N-labeled hRpn13 Pru with DMSO (vehicle control, black) or 2-fold molar excess of **XL44** derivatives **XL52 - XL56** (orange). Spectra were acquired at 600 MHz and 10°C. In the first panel, arrows highlight shifting of hRpn13 Pru signals from their apo state to their **XL52**-bound state. Residue signals that disappear (italicized in grey) or appear (red) after **XL52** addition are labeled. In other panels, some **XL44**-binding hRpn13 residues (Met31, Leu33, Lys34, Cys88, Phe106 and Trp108) are labeled. hRpn13 Val38 is labeled in red when present in the spectra.

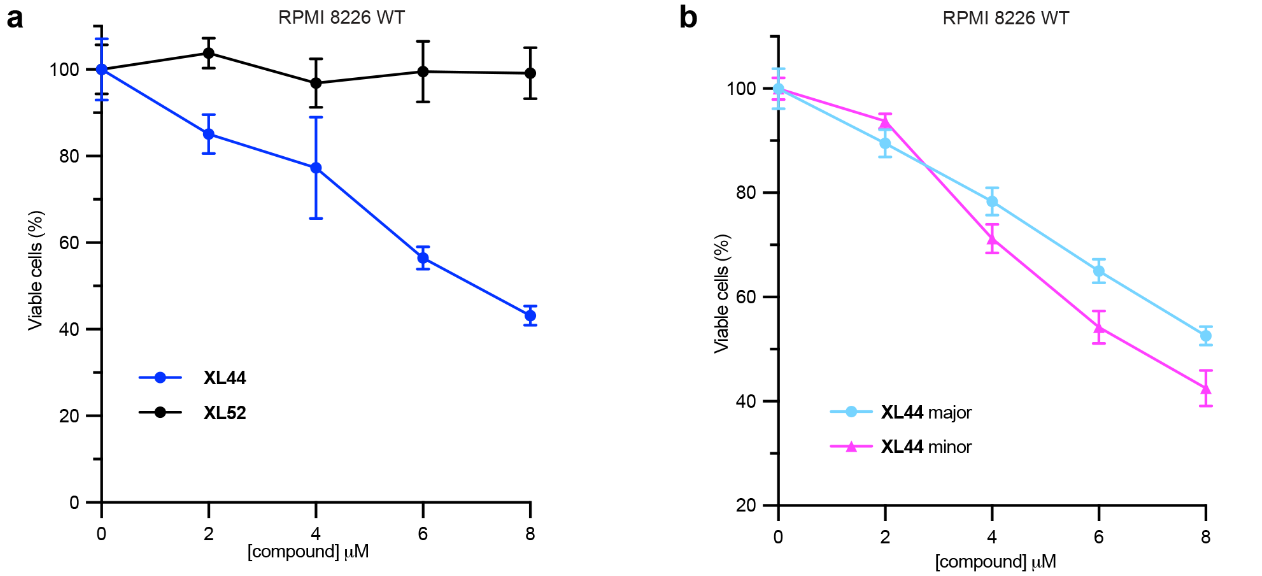

Supplementary Fig. 6 MTT assay of RPMI 8226 cells treated with XL44, XL52 or XL44 stereoisomers. RPMI 8226 cells were treated with varying concentrations of **XL44** (**a**, blue), **XL52** (**a**, black), **XL44** major (**b**, light blue) or minor (**b**, pink) stereoisomer (as defined in Supplementary Fig. 1) for 48 hours and cell metabolism measured by an MTT assay; data represent mean ± SD of n = 6 biological replicates. Viability is calculated as $(\lambda_{570})_{sample}/(\lambda_{570})_{control}$*100 (%).

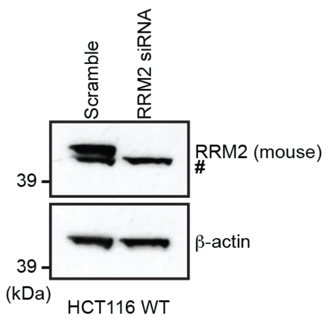

Supplementary Fig. 7 Only the upper band detected by anti-RRM2 mouse antibodies is reduced by siRNA knockdown of RRM2. Immunoblots for RRM2 (mouse) and β-actin (a loading control) of lysates extracted from HCT116 cells transfected with 50 nM scramble (control) or RRM2 siRNA for 48 hours. A black pound "#" indicates an unspecific band detected by the RRM2 antibodies (mouse).

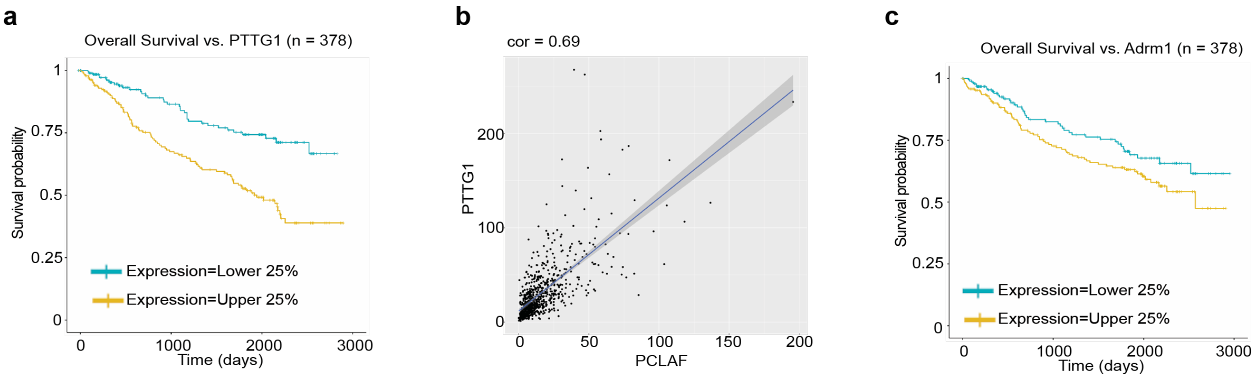

Supplementary Fig. 8 Correlation of expression level with overall survival in myeloma patients for PTTG1 and hRpn13. **a**, **c**, Graphical depiction of overall survival for myeloma patients with high (yellow) or low (blue) expression of PTTG1 (**a**, p-value < 0.0001) or hRpn13-expressing Adrm1 (**c**, p-value = 0.07). **b**, Graphical depiction of correlation of PCLAF and PTTG1 gene expression levels in myeloma patients.

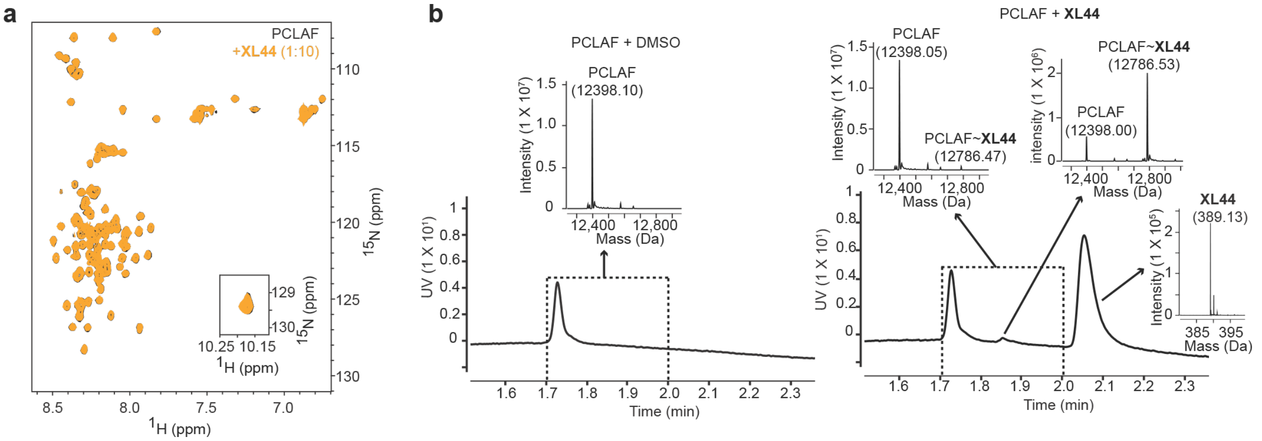

Supplementary Fig. 9 XL44 does not bind to PCLAF. **a**, ^{1}H, ^{15}N HSQC spectra of 20 μM PCLAF (black) and with 10-fold molar excess of **XL44** (orange) in NMR buffer. The spectra were recorded at 700 MHz and 25°C. **b**, 4 μM purified PCLAF (MW: 12397.95 g/mol) was incubated with DMSO (a vehicle control, left panel) or 40 μM **XL44** (right panel) for 2 hours at 4°C and the samples subjected to LC-MS analysis to detect the formation of **XL44** adducts. UV spectra are displayed for each sample in **b**. Samples eluted between 1.74 and 2.01 minutes (dashed rectangle) were analyzed to generate MS spectra as indicated.

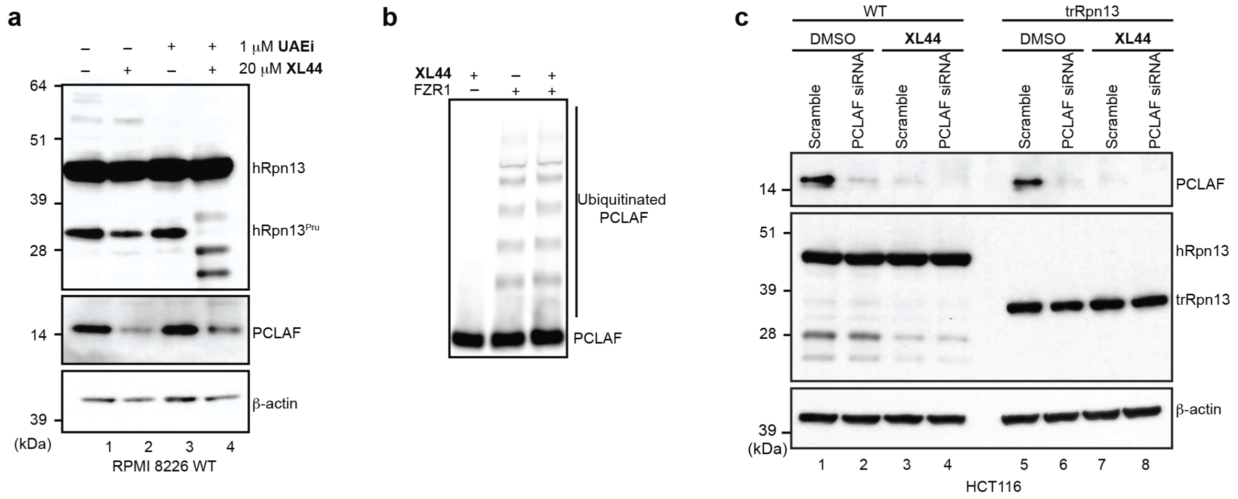

Supplementary Fig. 10 XL44 depletion of PCLAF does not require ubiquitin or hRpn13. a, Lysates from RPMI 8226 WT cells treated for 24 hours with 1 μM UAE inhibitor **MLN4924** (**UAEi**), 20 μM **XL44**, 1 μM **MLN4924** (**UAEi**) and 20 μM **XL44** (co-treatment), or DMSO (vehicle control) were immunoprobed for hRpn13, PCLAF and β-actin. **b**, Ubiquitination of the fluorescently labeled PCLAF by APC/C^{FZR1} without or with **XL44** was monitored by scanning of an SDS-PAGE gel. **c**, Immunoblots of PCLAF, hRpn13 and β-actin (a loading control) from cell lysates extracted from HCT116 WT or trRpn13 cells transfected for 48 hours with 50 nM scramble (control) or PCLAF siRNA and then treated for 24 hours with 20 μM **XL44** or DMSO (vehicle control).

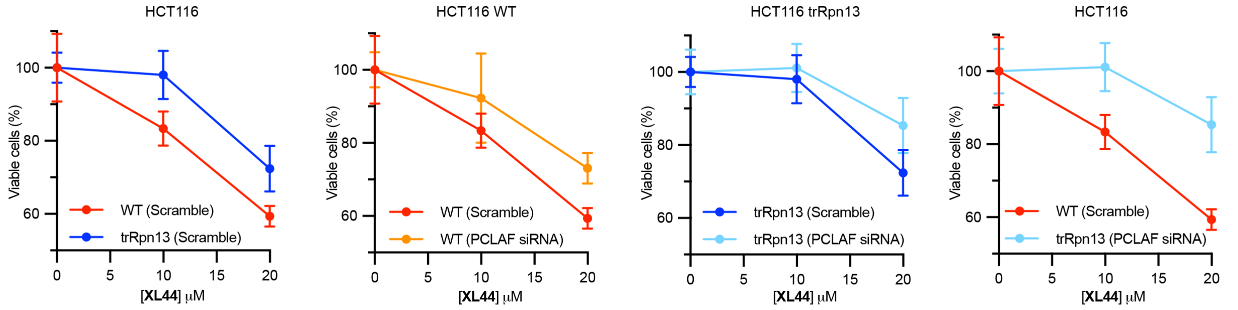

Supplementary Fig. 11 XL44 activity is impaired by dual knockdown of PCLAF and hRpn13. Cell metabolism measured by an MTT assay for HCT116 WT or trRpn13 cells transfected for 48 hours with 50 nM scramble (WT and trRpn13 in red and blue, respectively) or PCLAF (WT and trRpn13 in orange and light blue, respectively) siRNA and subsequently treated for 24 hours with DMSO (vehicle control) or 10 μM or 20 μM **XL44**. Data represent mean ± SD of n = 6 biological replicates. Viability is calculated as $(\lambda_{570})_{sample}/(\lambda_{570})_{control}$*100 (%).

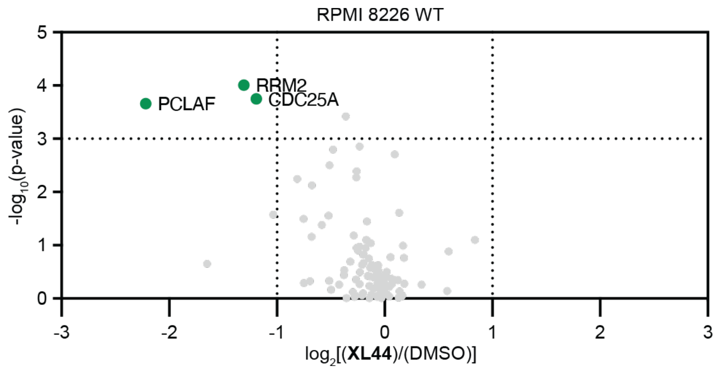

Supplementary Fig. 12 Among APC/C substrates detected by TMT-MS, only PCLAF, RRM2, and CDC25A were reduced in XL44-treated RPMI 8226 cells. Volcano plot extracted from Fig. 6a of proteomic changes for putative APC/C substrates, plotted and colored as in Fig. 6a.

Supplementary Table 1. Nomenclature and docking score for an 18-compound library generated based on chemical similarity to XL5.

Chemical structure	Compound	Nomenclature	Docking score (Schrödinger)
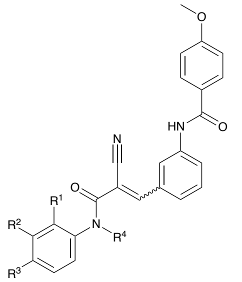	R^1: F; R^2, R^3, R^4: H	**XL34**	-5.474
	R^3: CH_3; R^1, R^2, R^4: H	**XL35**	-5.765
	R^2: OCH_3; R^1, R^3, R^4: H	**XL36**	-4.683
	R^1: CH_3; R^2, R^3, R^4: H	**XL37**	-4.801
	R^3: F; R^1, R^2, R^4: H	**XL38**	-3.937
	R^1, R^2, R^3, R^4: H	**XL39**	-1.173
	R^2: CH_3; R^1, R^3, R^4: H	**XL40**	-4.909
	R^3: OCH_3; R^1, R^2, R^4: H	**XL41**	-4.519
	R^4: CH_3; R^1, R^2, R^3: H	**XL42**	-5.510
	R^1: OCH_3; R^2, R^3, R^4: H	**XL43**	-4.747
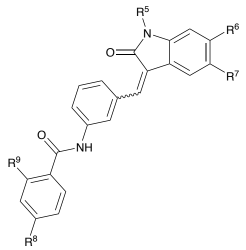	R^7: F; R^5, R^6, R^9: H; R^8: OCH_3	**XL44**	**-6.525**
	R^5, R^6, R^7, R^9: H; R^8: OCH_3	**XL45**	-5.514
	R^5: CH_3; R^6, R^7, R^9: H; R^8: OCH_3	**XL46**	-5.516
	R^6: CN; R^5, R^7, R^8: H; R^9: Cl	**XL47**	-5.488
	R^6: CN; R^5, R^7, R^9: H; R^8: OCH_3	**XL48**	-5.001
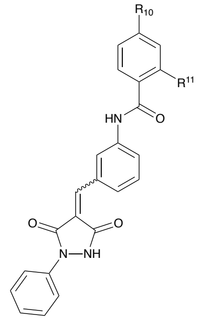	R^{10}: OCH_3, R11: H	**XL49**	-4.730
	R^{10}: Cl, R11: H	**XL50**	-4.545
	R^{10}: H, R11: Cl	**XL51**	-4.450

Supplementary Table 2. List of interactions between XL44 and hRpn13 Pru in the A-chain and B-chain.

A-chain				B-chain			
Protein		XL44		Protein		XL44	
Residue no.	atom name	atom name	Distance (Å)	Residue no.	atom name	atom name	Distance (Å)
M31	Cβ	F1	3.62	M31	Cγ	F1	3.31
M31	Cγ	F1	3.14	M31	Sδ	F1	3.82
M31	Sδ	F1	3.64	M31	Cε	C1	3.75
M31	Cε	C1	3.67	M31	Cε	C2	3.83
M31	Cε	C2	3.87	M31	Cε	O2	3.9
M31	Cε	C18	3.73	M31	Cε	C16	3.79
M31	Cε	F1	2.97	M31	Cε	C18	3.86
S32	C	F1	3.78	M31	Cε	F1	3.14
S32	O	F1	3.75	S32	C	F1	3.74
L33	Cδ2	N1	3.71	S32	O	F1	3.77
L33	Cδ2	C4	3.47	L33	N	F1	3.85
L33	Cδ2	C5	3.57	L33	Cδ2	N1	3.82
V38	Cγ1	C2	3.73	L33	Cδ2	C3	3.86
V38	Cγ1	N2	3.23	L33	Cδ2	C4	3.47
V38	Cγ1	C14	3.37	L33	Cδ2	C5	3.5
V38	Cγ1	C15	3.61	V38	Cγ1	N2	3.22
V38	Cγ2	C2	3.8	V38	Cγ1	C13	3.85
P40	Cβ	C19	3.86	V38	Cγ1	C14	3.26
P40	Cβ	C20	3.87	V38	Cγ1	C15	3.54
K42	Nζ	C23	3.53	V38	Cγ2	C2	3.88
V85	Cγ1	N1	3.62	P40	Cβ	C19	3.76
Q87	Cβ	O1	3.13	P40	Cβ	C20	3.81
Q87	Nε2	O1	3.77	P40	Cβ	C23	3.66
C88	N	C9	3.77	P40	Cγ	C20	3.87
C88	Cα	C9	3.48	P40	Cγ	C21	3.83
C88	Cα	C10	3.84	P40	Cγ	C23	3.59
C88	Cβ	C9	3.15	K42	Nζ	C23	3.46
C88	Cβ	C10	3.56	V85	Cγ1	N1	3.74
C88	Cβ	C15	3.73	Q87	C	O1	3.86
C88	Sγ	O1	3.82	Q87	Cβ	O1	3.05
C88	Sγ	C2	3.77	C88	Cα	C9	3.37
C88	Sγ	C3	3.21	C88	Cα	C10	3.87
C88	Sγ	C4	3.85	C88	Cβ	C9	3.27
C88	Sγ	C7	2.83	C88	Cβ	C10	3.8
C88	Sγ	C8	3.31	C88	Sγ	N1	3.84
C88	Sγ	C9	1.79	C88	Sγ	C2	3.76

C88	Sγ	C10	2.68	C88	Sγ	C3	3.23
C88	Sγ	C11	3.85	C88	Sγ	C4	3.73
C88	Sγ	C15	3.03	C88	Sγ	C7	3.04
V93	Cγ2	O2	3.54	C88	Sγ	C8	3.39
F106	Cγ	C5	3.87	C88	Sγ	C9	2.23
F106	Cγ	C6	3.37	C88	Sγ	C10	3.18
F106	Cδ1	C5	3.35	C88	Sγ	C15	3.46
F106	Cδ1	C6	3.32	P89	Cδ	C10	3.67
F106	Cδ2	C6	3.69	P89	Cδ	C11	3.42
F106	Cε1	C5	3.23	P89	Cδ	C12	3.61
F106	Cε1	C6	3.67	V93	Cγ2	O2	3.63
F106	Cζ	C5	3.58	F106	Cγ	C6	3.47
W108	Cγ	C19	3.8	F106	Cδ1	C5	3.61
W108	Cδ2	C19	3.84	F106	Cδ1	C6	3.37
				F106	Cδ2	C6	3.77
				F106	Cε1	C5	3.36
				F106	Cε1	C6	3.57
				F106	Cζ	C5	3.68
				F106	Cζ	C6	3.83
				W108	Cδ2	C19	3.83

Supplementary Table 3. Parallel Artificial Membrane Permeability Assay (PAMPA) for XL44 and XL52.

Compound	Permeability (10^{-6} cm/s)
**XL44**	92.4
**XL52**	<0.01

Supplementary Note 1

General information for chemical synthesis. Starting materials were used as received unless otherwise noted. All moisture sensitive reactions were performed in an inert atmosphere of argon with oven dried glassware. Reagent grade solvents were used for extractions and flash chromatography. Reaction progress was monitored by LC-MS analysis performed on an Agilent UPLC/MS instrument equipped with a RP-C18 column (Poroshell 120 SB-C18, 4.6 X 50 mm, 2.7 µm or Zorbax 300SB-C18, 4.6 X 50 mm, 3.5 µm), dual atmospheric pressure chemical ionization (APCI)/electrospray (ESI) mass spectrometry detector, and photodiode array detector. Flash chromatography was performed by using a RediSepRf NP-silica (40-63 µm 60 Å) or a Teledyne RediSepRf Gold RP-C18 column (20-40 µm 100 Å) in a Teledyne ISCO CombiFlash Rf 200 purification system unless otherwise specified. ^{1}H NMR spectra were recorded on an Agilent 400 MHz or Bruker 800 MHz spectrometer and are reported in parts per million (ppm) on the δ scale relative to $CDCl_3$ (δ 7.26) and DMSO-d_6 (δ 2.50) as internal standards. Data are reported as follows: chemical shift, multiplicity (s = singlet, d = doublet, t = triplet, q = quartet, b = broad, m = multiplet), coupling constants (Hz), and integration. ^{13}C-NMR spectra were recorded on an Agilent 100 MHz or Bruker 200 MHz spectrometer and are reported in parts per million (ppm) on the δ scale relative to $CDCl_3$ (δ 77.00) and DMSO-d_6 (δ 39.52). Note: The recorded ^{1}H NMRs of $^{13}C_6$ labeled **XL44-$^{13}C_6$-CB** 1H NMR is very complex and difficult to interpret due to large couplings between proton and ^{13}C-carbon. To remove the large couplings of H-^{13}C, the BilevelDec ^{1}H NMR

method was used for $^{13}C_6$ labeled XL44 compound and both the ^1H NMR and BilevelDec ^1H NMR data are reported.

Synthesis and characterization data. XL5B, XL44B, XL44-$^{13}C_6$-CB, XL52, XL53, XL54, XL55, and XL56 were synthesized according to the procedures described below and characterization data (^1H NMR, ^{13}C NMR, ^{19}F NMR and high-resolution mass spectrometry (HRMS)) are included.

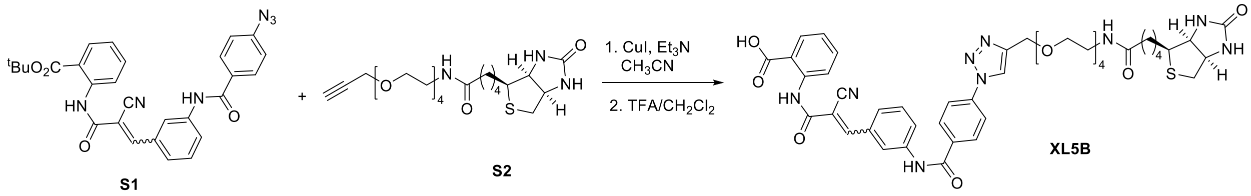

To a stirred solution of azide[1] **S1** (30 mg, 0.06 mmol) in CH_3CN (5 mL), biotin-PEG4-alkyne **S2** (33 mg, 0.06 mmol), CuI (2.6 mg, 0.012 mmol), and Et_3N (9 µL, 0.06 mmol) were added under argon atmosphere. The reaction mixture was stirred for overnight at room temperature and then solvent was removed by rotary evaporator. Saturated aqueous NH_4Cl (3 mL) and CH_2Cl_2 (5 mL) were added to the crude mixture and stirred for 5 min. Then organic layer was separated, and the aqueous layer was extracted with CH_2Cl_2 (5 x 5 mL). The combined organic layers were dried over Na_2SO_4, filtered and concentrated to afford the crude material. The crude product was dissolved in 20 mol% TFA in CH_2Cl_2 (1 mL) and stirred for 3 hours at room temperature. After the deprotection was completed, the solvent was removed and purified by a preparatory HPLC with a XBridge BEH C18 OBD Prep Column, 130Å, 5 µm, 30 mm X 150 mm reverse-phase

column as the stationary phase. Water (buffered with 0.05% trifluoroacetic acid) and MeCN were used as the mobile phase and HPLC conditions: UV collection 254 nm, flow rate 30 mL/min, 20% MeCN as linear gradient for 5 min and 20% → 65% MeCN for 5 to 20 min. The HPLC fractions were combined and lyophilized to yield **XL5B** (28 mg, 52%, single isomer). ^{1}H NMR (400 MHz, dmso) δ 12.24 (s, 1H), 10.67 (s, 1H), 8.94 (s, 1H), 8.62 (dd, *J* = 8.4, 1.1 Hz, 1H), 8.49 (t, *J* = 2.0 Hz, 1H), 8.40 (s, 1H), 8.23 – 8.17 (m, 2H), 8.14 – 8.09 (m, 2H), 8.05 (dd, *J* = 7.9, 1.7 Hz, 1H), 7.97 (ddd, *J* = 8.2, 2.1, 1.0 Hz, 1H), 7.83 – 7.76 (m, 2H), 7.68 (ddd, *J* = 8.6, 7.3, 1.6 Hz, 1H), 7.60 (t, *J* = 8.0 Hz, 1H), 7.31 – 7.22 (m, 1H), 6.39 (s, 1H), 6.33 (s, 1H), 4.64 (s, 2H), 4.27 (dd, *J* = 7.8, 5.0 Hz, 1H), 4.10 (ddd, *J* = 7.8, 4.4, 1.8 Hz, 1H), 3.67 – 3.59 (m, 2H), 3.60 – 3.54 (m, 2H), 3.52 – 3.44 (m, 8H), 3.36 (t, *J* = 5.9 Hz, 2H), 3.15 (q, *J* = 5.8 Hz, 2H), 3.06 (ddd, *J* = 8.6, 6.2, 4.4 Hz, 1H), 2.79 (dd, *J* = 12.4, 5.1 Hz, 1H), 2.55 (d, *J* = 12.4 Hz, 1H), 2.04 (t, *J* = 7.4 Hz, 2H), 1.65 – 1.36 (m, 4H), 1.28 (dq, *J* = 14.1, 7.0 Hz, 2H). ^{13}C NMR (101 MHz, dmso) δ 172.56, 170.22, 165.13, 163.14, 159.45, 153.23, 145.91, 140.64, 140.25, 139.26, 134.78, 134.67, 132.60, 131.69, 130.17, 130.06, 126.34, 125.35, 124.29, 122.79, 122.48, 120.86, 120.07, 117.52, 115.98, 106.66, 70.27, 70.25, 70.17, 70.01, 69.66, 69.61, 63.87, 61.48, 59.63, 55.86, 40.29, 38.89, 35.54, 28.63, 28.48, 25.70. HRMS (m/z): $[M+H]^+$ calcd. for $C_{45}H_{52}N_9O_{10}S$, 910.3558; found, 910.3541.

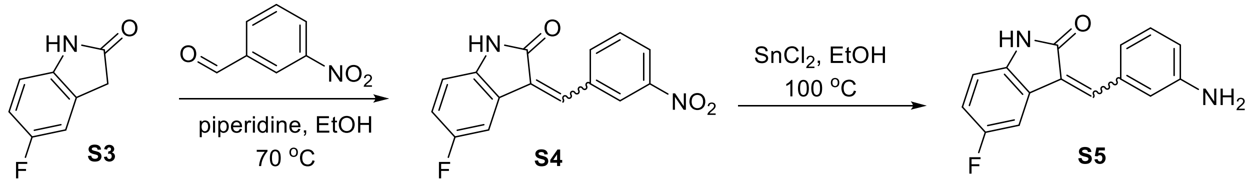

In a thick-walled vial, 5-fluoroindolin-2-one **S3** (1 g, 6.6 mmol), and 3-nitrobenzaldehyde (1 g, 6.6 mmol) were dissolved in EtOH (15 mL) and then piperidine (0.2 mL, 2 mmol) was added at room temperature. The vial was sealed and heated at 70 ^{o}C for 6 hours. The yellow solid precipitate was collected, washed with cold EtOH (2 x 20 mL) and dried under vacuum to afford yellow solid **S4** (1.3 g, 70%). ^{1}H NMR (400 MHz, dmso) δ 10.75 (s, 1H), 9.38 (t, *J* = 2.0 Hz, 1H), 8.74 – 8.51 (m, 1H), 8.28 (dd, *J* = 7.9, 2.3 Hz, 1H), 8.02 (s, 1H), 7.75 (t, *J* = 8.0 Hz, 1H), 7.65 (dd, *J* = 8.9, 2.6 Hz, 1H), 7.07 (td, *J* = 9.1, 2.6 Hz, 1H), 6.81 (dd, *J* = 8.5, 4.4 Hz, 1H). ^{13}C NMR (101 MHz, dmso) δ 167.41, 158.42 (d), 148.13, 138.45, 137.97 (d), 136.01, 135.52, 130.24, 129.27 (d), 126.46, 126.23 (d), 125.22, 116.50 (d), 110.92 (d), 108.29 (d). ^{19}F NMR (376 MHz, dmso) δ -122.21. HRMS (m/z): $[M+H]^{+}$ calcd. for C15H10FN2O3, 285.0675; found, 285.0666.

Nitro compound **S4** (500 mg, 1.76 mmol) and $SnCl_4$ (1 g, 5.28 mmol) were dissolved in EtOH (20 mL) and then refluxed for 4 hours. To the cooled reaction mixture, saturated aqueous potassium fluoride (10 mL) was added and stirred for 1 hour. The crude product was extracted with EtOAc (3 x 20 mL), and dried over anhydrous Na_2SO_4. After concentration, the crude product was purified by an ISCO combi flash silica gel column (EtOAc/hexanes) to provide brown colored aniline derivative **S5** (295 mg, 66%, mixture of isomers E/Z = 5:1). Major isomer: ^{1}H NMR (400 MHz, DMSO) δ 10.60 (s, 1H), 7.58 (s, 1H), 7.34 (dd, *J* = 9.4, 2.7 Hz, 1H), 7.19 (t, *J* = 7.8 Hz, 1H), 7.07 (ddd, *J* = 9.4, 8.5, 2.7 Hz, 1H), 6.88 – 6.82 (m, 2H), 6.79 (dd, *J* = 7.5, 0.9 Hz, 1H), 6.72 – 6.67 (m, 1H), 5.37 (s, 2H). ^{13}C NMR (101 MHz, DMSO) δ 169.24, 157.62 (d), 149.55, 139.55 (d), 139.19, 134.98, 129.90, 127.16 (d), 122.55 (d), 117.21, 116.57 (d), 116.12, 114.13, 111.11 (d),

110.24 (d). ^{19}F NMR (376 MHz, DMSO) δ -122.09 (td). Selected for minor isomer: ^{1}H NMR (400 MHz, DMSO) δ 10.56 (s, 1H), 7.71 (s, 1H), 7.64 (dd, *J* = 9.1, 2.7 Hz, 1H), 7.61 (s, 1H), 7.13 (t, *J* = 7.8 Hz, 1H), 7.01 (ddd, *J* = 9.6, 8.4, 2.6 Hz, 1H), 5.18 (s, 2H). ^{13}C NMR (101 MHz, DMSO) δ 167.52, 157.41 (d), 148.90, 140.28, 137.22, 134.72, 129.11, 127.21, 126.05 (d), 120.97, 117.65, 117.34, 115.20 (d), 110.30, 107.59 (d). ^{19}F NMR (376 MHz, DMSO) δ -122.65 (td). HRMS (m/z): $[M+H]^+$ calcd. for $C_{15}H_{12}FN_2O$, 255.0934; found, 255.0938.

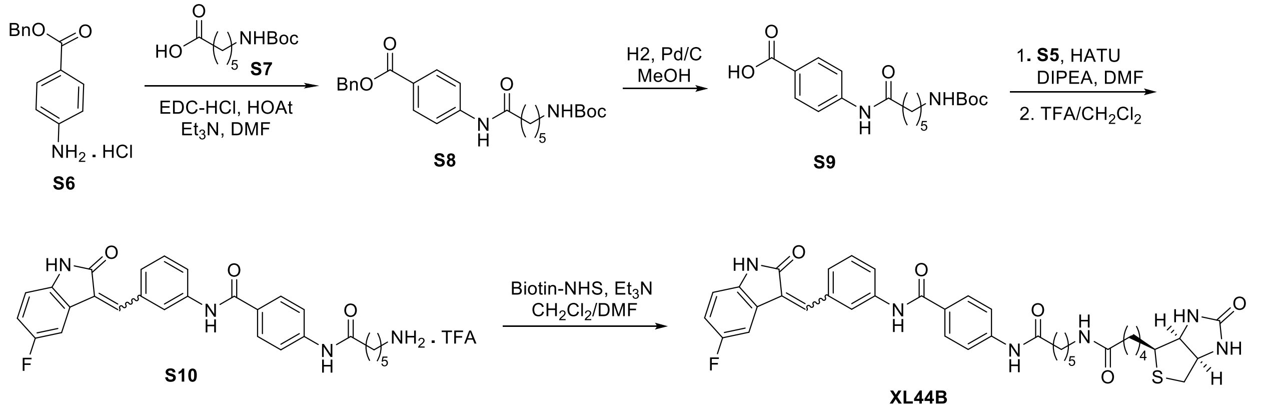

To a stirred solution of boc-6-Ahx-OH **S7** (210 mg, 0.91 mmol) in DMF (10 mL), benzyl 4-aminobenzoate hydrochloride **S6** (240 mg, 0.91 mmol), N-(3-dimethylaminopropyl)-N′-ethylcarbodiimide hydrochloride (EDC-HCl) (280 mg, 1.46 mmol), 1-hydroxy-7-azabenzotriazole (HOAt) (178 mg, 1.46 mmol), and Et_3N (0.46 mL, 3.2 mmol) were added at room temperature and stirred for overnight. Water (20 mL) was added and extracted with EtOAc (30 mL). The organic layer was washed with aqueous NaCl (2 x 20 mL) and dried over anhydrous Na_2SO_4. After concentration, the crude product was purified by an ISCO combi flash silica gel column (EtOAc/Hexanes) to afford **S8** (165 mg, 44%). ^{1}H NMR (400 MHz, cdcl$_3$) δ 8.24 (s, 1H), 8.05 – 7.96 (m, 2H), 7.69 – 7.61 (m, 2H), 7.46 –

7.28 (m, 5H), 5.33 (s, 2H), 4.69 (s, 1H), 3.08 (q, *J* = 6.7 Hz, 2H), 2.35 (t, *J* = 7.5 Hz, 2H), 1.71 (p, *J* = 7.6 Hz, 2H), 1.53 – 1.44 (m, 2H), 1.42 (s, 8H), 1.40 – 1.28 (m, 2H). ^{13}C NMR (101 MHz, cdcl$_3$) δ 171.83, 166.10, 156.23, 142.72, 136.08, 130.88, 128.58, 128.22, 128.10, 125.13, 118.81, 79.26, 66.59, 40.22, 37.43, 29.70, 28.43, 26.21, 24.91. HRMS (m/z): [M+Na]$^+$ calcd. for $C_{25}H_{32}N_2O_5Na$, 463.2209; found, 463.2199.

To a methanolic solution of **S8** (150 mg, 0.34 mmol), palladium on carbon (4 mg) was added under inert atmosphere. The resulting slurry was purged with argon (1 min) before H_2 gas was added via a double walled balloon. Upon completion as indicated by LC-MS analysis, the reaction mixture was purged with nitrogen (2 min) and filtered through a pad of celite with the aid of MeOH. The concentrated crude product **S9** was dried and used for the next step without further purification. ^{1}H NMR (400 MHz, DMSO) δ 10.17 (s, 1H), 7.87 (d, *J* = 8.3 Hz, 2H), 7.69 (d, *J* = 8.3 Hz, 2H), 6.77 (t, *J* = 5.9 Hz, 1H), 2.91 (q, *J* = 6.6 Hz, 2H), 2.33 (t, *J* = 7.4 Hz, 2H), 1.59 (p, *J* = 7.5 Hz, 2H), 1.45 – 1.38 (m, 2H), 1.36 (s, 9H), 1.33 – 1.21 (m, 2H). ^{13}C NMR (101 MHz, DMSO) δ 172.18, 167.60, 156.05, 143.56, 130.74, 126.08, 118.64, 77.76, 40.24, 36.92, 29.79, 28.74, 26.45, 25.21. HRMS (m/z): [M+Na]$^+$ calcd. for $C_{18}H_{26}N_2O_5Na$, 373.1739; found, 373.1726.

The benzoic acid **S9** (62 mg, 0.18 mmol) was dissolved in DMF (3 mL), aniline **S5** (49 mg, 0.19 mmol), 1-[bis(dimethylamino)methylene]-1H-1,2,3-triazolo[4,5-b]pyridinium 3-oxid hexafluorophos-phate (HATU) (75 mg, 0.19 mmol), and DIPEA (83 µL, 0.45 mmol) were added at room temperature and the mixture was stirred for overnight. Water (5 mL) was added and extracted with EtOAc (20 mL). The organic layer was washed with

aqueous NaCl (2 x 10 mL) and dried over anhydrous Na_2SO_4. After concentration, the crude product was purified by an ISCO combi flash silica gel column (CH_2Cl_2/MeOH). The purified material was subjected to 20 mol% TFA in CH_2Cl_2 (1 mL) and stirred for 3 hours at room temperature. Upon completion of Boc deprotection as indicated by LC-MS analysis, the solvent was removed and lyophilized to afford TFA salt of **S10** (70 mg, 68% over two steps), which was used for the next step without further purification. TFA salt of **S10** (35 mg, 0.072 mmol) was dissolved in 3 mL of CH_2Cl_2/DMF (1:1), and then biotin-NHS (21 mg, 0.079 mmol) and Et_3N (52 µL, 0.36 mmol) were added at room temperature under argon atmosphere. The reaction mixture was stirred for overnight, water (10 mL) was added and then stirring was continued for 30 minutes. The yellow precipitate was collected and washed thoroughly with water (30 mL) and dried under vacuum to afford pure **XL44B** (48 mg, 94%, *E*/*Z* = 6:1). Major isomer: ^{1}H NMR (400 MHz, dmso) δ 10.63 (s, 1H), 10.30 (s, 1H), 10.14 (s, 1H), 8.20 (s, 1H), 7.94 – 7.90 (m, 2H), 7.82 – 7.77 (m, 1H), 7.74 – 7.70 (m, 3H), 7.67 (s, 1H), 7.51 (t, *J* = 7.9 Hz, 1H), 7.45 (dd, *J* = 9.4, 2.6 Hz, 1H), 7.38 (d, *J* = 7.6 Hz, 1H), 7.08 (td, *J* = 9.0, 2.6 Hz, 1H), 6.85 (dd, *J* = 8.5, 4.6 Hz, 1H), 6.40 (s, 1H), 6.34 (s, 1H), 4.27 (dd, *J* = 7.7, 5.1 Hz, 1H), 4.13 – 4.07 (m, 1H), 3.11 – 2.98 (m, 4H), 2.79 (dd, *J* = 12.5, 5.1 Hz, 1H), 2.55 (d, *J* = 12.4 Hz, 1H), 2.33 (t, *J* = 7.4 Hz, 3H), 2.02 (t, *J* = 7.4 Hz, 2H), 1.63 – 1.53 (m, 3H), 1.50 – 1.35 (m, 4H), 1.33 – 1.22 (m, 5H). ^{13}C NMR (101 MHz, dmso) δ 172.25, 172.14, 169.07, 165.63, 163.15, 157.72 (d, *J* = 235.2 Hz), 142.93, 140.12, 139.76, 137.78, 134.81, 129.78, 129.13, 129.07, 127.90 (d, *J* = 2.9 Hz), 125.35, 122.29, 122.19 (d, *J* = 8.9 Hz), 120.77, 118.63, 116.95 (d, *J* = 23.7 Hz), 111.14 (d, *J* = 8.0 Hz), 110.71 (d, *J* = 25.9 Hz), 61.48, 59.63, 55.88, 40.29, 38.72, 36.86, 35.67, 29.47, 28.66, 28.48, 26.57, 25.79, 25.16. ^{19}F NMR (376 MHz, dmso) δ -121.90 (td).

Selected for minor isomer: ^{1}H NMR (400 MHz, dmso) δ 10.59 (s, 1H), 10.24 (s, 1H), 8.50 (s, 1H), 8.25 (d, *J* = 7.8 Hz, 1H), 7.86 (s, 1H), 6.78 (dd, *J* = 8.5, 4.4 Hz, 1H), 3.15 (d, *J* = 5.3 Hz, 1H). ^{19}F NMR (376 MHz, dmso) δ -122.50 (td, *J* = 9.2, 4.3 Hz). HRMS (m/z): $[M+H]^+$ calcd. for $C_{38}H_{42}FN_6O_5S$, 713.2921; found, 713.2897.

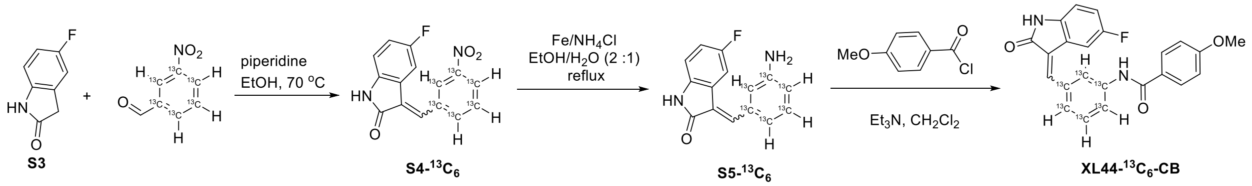

S3 S4-$^{13}C_6$ S5-$^{13}C_6$ XL44-$^{13}C_6$-CB

In a thick-walled vial, 5-fluoroindolin-2-one **S3** (74 mg, 0.49 mmol) and 3-nitro-benz-$^{13}C_6$-aldehyde[1] (77 mg, 0.49 mmol) were dissolved in EtOH (3 mL) and then piperidine (48 μL, 0.49 mmol) was added at room temperature. The vial was sealed and heated at 70 °C for 6 hours. The yellow solid product was collected, washed with cold EtOH (2 x 5 mL) and dried under vacuum to afford **S4-$^{13}C_6$** (95 mg, 67%). To a stirred solution of **S4-$^{13}C_6$** (85 mg, 0.29 mmol) in 30 mL EtOH/H_2O (2:1), iron (82 mg, 1.45 mmol) and NH_4Cl (8 mg, 0.15 mmol) were added at room temperature. The reaction mixture was refluxed for 4 days by monitoring the reaction by LCMS. The reaction mixture cooled to room temperature and filtered through a thick pad of celite with the aid of CH_2Cl_2/MeOH (9:1). The filtrate was transferred to separating funnel, organic layer was separated and then aqueous layer was extracted with CH_2Cl_2 (2 x 10 mL). The combined organic layers were dried over anhydrous Na_2SO_4, and concentrated to provide **S5-$^{13}C_6$**, which was used for the next step without further purification. The crude **S5-$^{13}C_6$** (46 mg, 0.17 mmol) was dissolved in CH_2Cl_2 (3 mL), and then 4-methoxybenzoyl chloride (26 μL, 0.19 mmol) and Et_3N (50 μL, 0.34 mmol) were added at room temperature. The reaction mixture stirred for overnight

and water (3 mL) was added. The organic layer was separated and the aqueous layer was extracted with CH_2Cl_2 (2 x 5 mL). Combined organic layers were dried, concentrated and purified by ISCO combi flash column chromatography to afford **XL44-^{13}C$_6$-CB** (6 mg, 9%). ^{1}H NMR (400 MHz, dmso) δ 10.65 (s, 1H), 10.29 (s, 1H), 8.44 – 8.00 (m, 1H), 7.99 – 7.93 (m, 2H), 7.76 – 7.55 (m, 3H), 7.47 (dd, *J* = 9.5, 2.6 Hz, 1H), 7.38 – 7.05 (m, 4H), 6.87 (dd, *J* = 8.5, 4.6 Hz, 1H), 3.84 (s, 3H). ^{1}H Bilevel Dec NMR (400 MHz, dmso) δ 10.64 (s, 1H), 10.29 (s, 1H), 8.22 (s, 1H), 8.02 – 7.92 (m, 2H), 7.82 (d, *J* = 7.8 Hz, 1H), 7.69 (s, 1H), 7.59 – 7.43 (m, 2H), 7.39 (d, *J* = 7.5 Hz, 1H), 7.15 – 7.02 (m, 3H), 6.87 (dd, *J* = 8.5, 4.6 Hz, 1H), 3.84 (s, 3H). ^{13}C NMR (101 MHz, dmso) δ 168.64, 168.58, 165.18, 162.04, 158.44, 156.10, 139.69 (td), 139.30, 134.32 (ddd), 129.68, 129.30 (td), 124.77 (td), 121.78 (dddd), 120.28 (ddd), 116.59, 116.36, 113.66, 110.67 , 110.38, 110.12, 55.45. ^{19}F NMR (376 MHz, dmso) δ -121.94 (td). HRMS (m/z): $[M+Na]^+$ calcd. for $C_{17}{}^{13}C_6H_{17}FN_2O_3Na$, 417.1322; found, 417.1318.

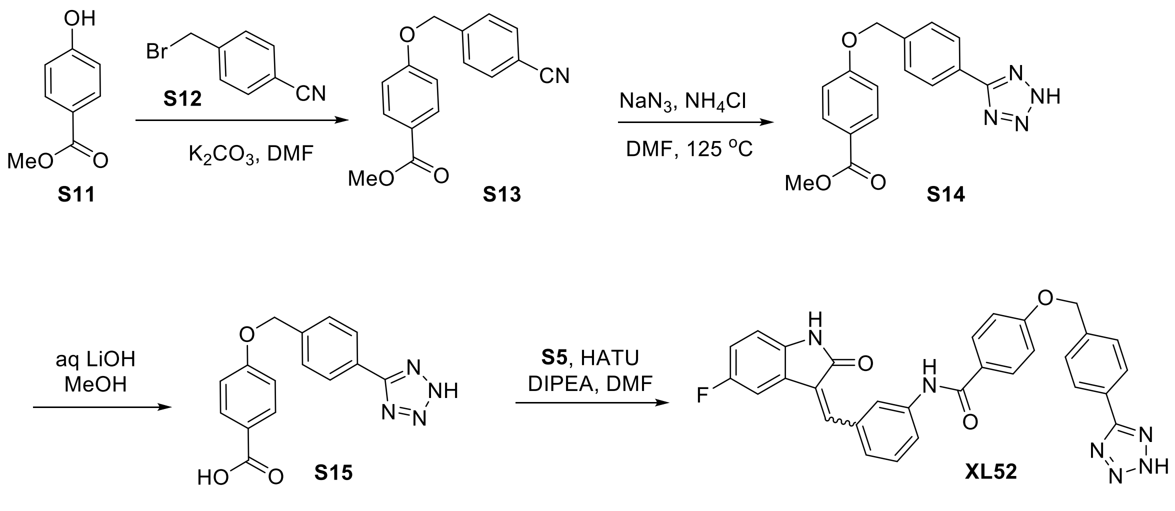

To a stirred solution of 4-cyanobenzyl bromide **S12** (1 g, 5.1 mmol) and methyl 4-hydroxybenzoate **S11** (815 mg, 5.3 mmol) in DMF (15 mL), K_2CO_3 (2.11 g, 15.3 mmol)

was added and stirred for 15 hours at room temperature. Water (30 mL) and EtOAc (50 mL) were added and then the organic layer was separated. The collected organic layer was washed with aqueous NaCl (2 x 30 mL) and dried over anhydrous Na_2SO_4. After concentration, the crude product was purified by an ISCO combi flash silica gel column (hexanes/EtOAc) to afford **S13** (white solid, 1.2 g, 88%). ^{1}H NMR (400 MHz, $CDCl_3$) δ 7.97 – 7.89 (m, 2H), 7.65 – 7.57 (m, 2H), 7.50 – 7.43 (m, 2H), 6.94 – 6.86 (m, 2H), 5.10 (s, 2H), 3.81 (s, 3H). ^{13}C NMR (101 MHz, $CDCl_3$) δ 166.65, 161.81, 141.71, 132.50, 131.74, 127.59, 123.44, 118.59, 114.41, 111.99, 68.94, 51.97. HRMS (m/z): $[M+H]^+$ calcd. for $C_{16}H_{14}NO_3$, 268.0974; found, 268.0968.

Nitrile **S13** (1 g, 3.74 mmol) was dissolved in DMF (20 mL) and then NaN_3 (365 mg, 5.61 mmol), and NH_4Cl (300 mg, 5.61 mmol) were added at room temperature under argon atmosphere. The reaction was heated at 125 ^{o}C for 16 hours and cooled to room temperature. Water (30 mL) was added to a cooled reaction mixture and extracted with EtOAc (50 mL). The organic layer was washed with brine (2 x 30 mL), dried (anhydrous Na_2SO_4), and purified the concentrated material by an ISCO combi flash silica gel column (CH_2Cl_2/MeOH) to provide **S14** (white solid, 625 mg, 54%). ^{1}H NMR (400 MHz, DMSO) δ 8.08 (d, *J* = 8.3 Hz, 2H), 7.94 (d, *J* = 8.9 Hz, 2H), 7.69 (d, *J* = 8.0 Hz, 2H), 7.21 – 7.14 (m, 3H), 5.31 (s, 2H), 3.82 (s, 3H). ^{13}C NMR (101 MHz, DMSO) δ 166.31, 162.50, 140.16, 132.95, 131.74, 128.95, 127.60, 124.38, 122.70, 115.35, 69.38, 52.33. HRMS (m/z): $[M+H]^+$ calcd. for $C_{16}H_{15}N_4O_3$, 311.1144; found, 311.1140.

To a stirred solution of **S14** (220 mg, 0.71 mmol) in 6 mL MeOH/THF (1:1), LiOH (85 mg, 3.55 mmol) in water (1 mL) was added at 0 °C. The reaction mixture was warmed to room temperature and stirred for 6 hours (monitored by LC-MS). Ice cold aqueous HCl (1M) was added to the reaction and then stirred for 30 minutes. The white precipitate was collected and washed with water (10 mL) and dried under vacuum to afford **S15** (196 mg, 93%). ^{1}H NMR (400 MHz, dmso) δ 12.63 (s, 1H), 8.05 (d, *J* = 8.0 Hz, 2H), 7.97 – 7.83 (m, 2H), 7.67 (d, *J* = 7.9 Hz, 2H), 7.11 (d, *J* = 8.8 Hz, 2H), 5.28 (s, 2H). ^{13}C NMR (101 MHz, dmso) δ 167.37, 162.18, 140.30, 132.90, 131.83, 128.92, 127.60, 123.82, 115.13, 69.30. HRMS (m/z): $[M+H]^+$ calcd. for $C_{15}H_{13}N_4O_3$, 297.0988; found, 297.0984.

Carboxylic acid **S15** (30 mg, 0.10 mmol) and **S5** (28 mg, 0.11 mmol) were dissolved in DMF (2 mL), HATU (42 mg, 0.11 mmol), and DIPEA (46 µL, 0.25 mmol) were then added at room temperature. The reaction stirred for overnight and purified by reverse-phase ISCO combi flash column chromatography. Water (buffered with 0.05% trifluoroacetic acid) and MeCN were used as the mobile phase, and the collected fractions were combined and lyophilized to provide **XL52** (5 mg, 9%, *E*/*Z* = 3:1). ^{1}H NMR (400 MHz, DMSO) δ 10.75 (s, 1H), 10.40 (s, 1H), 8.28 (s, 1H), 8.17 – 8.00 (m, 5H), 7.89 (d, *J* = 8.1 Hz, 1H), 7.78 – 7.66 (m, 3H), 7.60 – 7.40 (m, 3H), 7.28 – 7.20 (m, 2H), 7.15 (td, *J* = 8.9, 2.6 Hz, 1H), 6.93 (dd, *J* = 8.5, 4.6 Hz, 1H), 5.34 (s, 2H). ^{13}C NMR (101 MHz, DMSO) δ 169.10, 165.67, 161.46, 158.92, 156.58, 140.21, 139.80, 139.00, 138.68, 137.82, 134.82, 130.20, 129.77, 128.74, 127.93, 127.49, 127.21, 125.31, 122.30, 122.17, 120.79, 116.95 (d), 115.03, 111.22, 110.73 (d), 69.53. ^{19}F NMR (376 MHz, DMSO) δ -121.89 (td). HRMS (m/z): $[M+H]^+$ calcd. for $C_{30}H_{22}FN_6O_3$, 533.1737; found, 533.1735. Selected for minor

isomer: ^{1}H NMR (400 MHz, DMSO) δ 10.70 (s, 1H), 10.33 (s, 1H), 8.57 (s, 1H), 8.33 (d, *J* = 7.9 Hz, 1H), 7.93 (s, 1H), 7.11 – 7.04 (m, 1H), 6.86 (dd, *J* = 8.4, 4.4 Hz, 1H). ^{19}F NMR (376 MHz, DMSO) δ -122.47 (td). HRMS (m/z): [M+H]$^+$ calcd. for $C_{30}H_{22}FN_6O_3$, 533.1737; found, 533.1732.

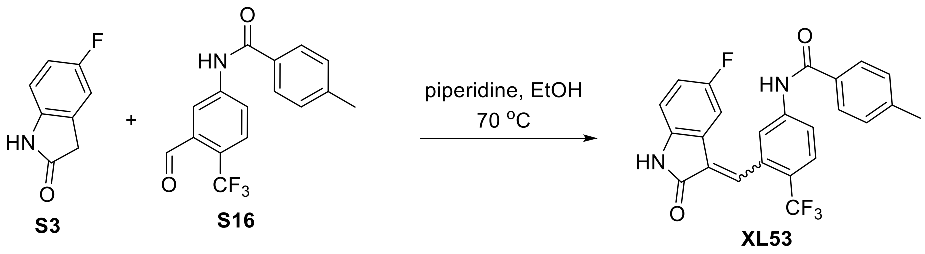

In a thick-walled vial, 5-fluoroindolin-2-one **S3** (16 mg, 0.11 mmol) and aldehyde **S16** (30 mg, 0.1 mmol) were dissolved in EtOH (5 mL) and then piperidine (10 μL, 0.1 mmol) was added at room temperature. The vial was sealed and heated at 70 °C for 6 hours (monitored by LC-MS). The yellow solid product was collected, washed with cold EtOH (2 x 5 mL) and dried under vacuum to afford yellow solid **XL53** (21 mg, 48%). ^{1}H NMR (400 MHz, dmso) δ 10.74 (s, 1H), 10.61 (s, 1H), 8.23 (d, *J* = 2.1 Hz, 1H), 8.09 (dd, *J* = 9.1, 2.1 Hz, 1H), 7.94 – 7.82 (m, 3H), 7.74 (d, *J* = 2.6 Hz, 1H), 7.34 (d, *J* = 8.0 Hz, 2H), 7.09 (td, *J* = 9.0, 2.6 Hz, 1H), 6.87 (dd, *J* = 8.6, 4.5 Hz, 1H), 6.77 (dd, *J* = 9.0, 2.6 Hz, 1H), 2.37 (s, 3H). ^{13}C NMR (101 MHz, dmso) δ 168.29, 166.45, 157.67 (d), 143.53, 142.74, 140.08 (d), 133.66 (d), 133.00, 131.70, 130.34, 129.48, 128.34, 128.11, 125.85, 123.14, 121.90 (d), 121.59 (d), 120.87 (d), 117.62 (d), 111.49 (d), 110.88 (d), 21.49. ^{19}F NMR (376 MHz, dmso) δ -58.46 (d), -121.77 (td). HRMS (m/z): [M+H]$^+$ calcd. for $C_{24}H_{17}F_4N_2O_2$, 441.1226; found, 441.1225.

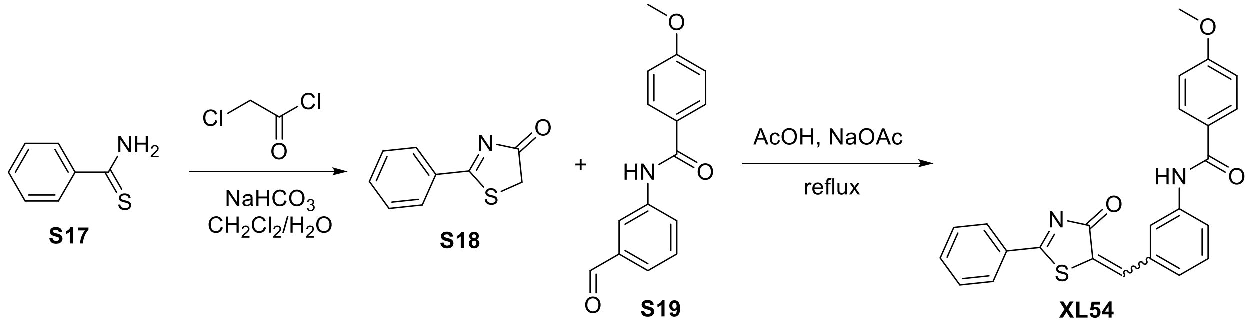

Intermediate **S18** was synthesized using the reported procedure.[2] To a stirred solution of **S17** (1 g, 7.3 mmol) in CH_2Cl_2 (40 mL), $NaHCO_3$ (1.23 g, 14.6 mmol) in 20 mL water was added. The reaction mixture cooled to 0 ^oC and then chloroacetyl chloride (0.58 mL, 7.3 mmol) was added. The reaction warmed to room temperature and stirred for overnight. The organic phase was separated, and aqueous phase was extracted with CH_2Cl_2 (2 x 50 mL). The combined organic layers were washed with aqueous NaCl (50 mL) and dried over anhydrous Na_2SO_4. The concentrated yellow crude product **S18** was used for the next step without further purification. In a thick-walled vial, aldehyde **S19** (20 mg, 0.08 mmol) in AcOH, thiazole **S18** (15 mg, 0.088 mmol), and NaOAc (3 mg, 0.024 mmol) were added. The vial was sealed and heated at 100 ^oC for 6 hours. The vial was cooled to room temperature and then yellow precipitate was collected by washing with cold ethanol (10 mL). The collected solid was purified by ISCO combi flash silica gel column chromatography (CH_2Cl_2/MeOH) to afford **XL54** (21 mg, 63%). ^1H NMR (400 MHz, dmso) δ 10.34 (s, 1H), 8.30 (t, J = 1.7 Hz, 1H), 8.23 – 8.16 (m, 2H), 8.05 – 7.96 (m, 3H), 7.95 – 7.87 (m, 1H), 7.86 – 7.78 (m, 1H), 7.74 – 7.65 (m, 2H), 7.62 – 7.46 (m, 2H), 7.14 – 7.00 (m, 2H), 3.84 (d, J = 0.6 Hz, 3H). ^{13}C NMR (101 MHz, dmso) δ 187.32, 182.57, 165.74, 162.55, 140.81, 138.46, 136.12, 134.12, 131.63, 130.22, 130.18, 128.99, 127.14, 126.97,

126.80, 123.64, 121.35, 114.13, 55.93. HRMS (m/z): $[M+H]^+$ calcd. for $C_{24}H_{19}N_2O_3S$, 415.1116; found, 415.1114.

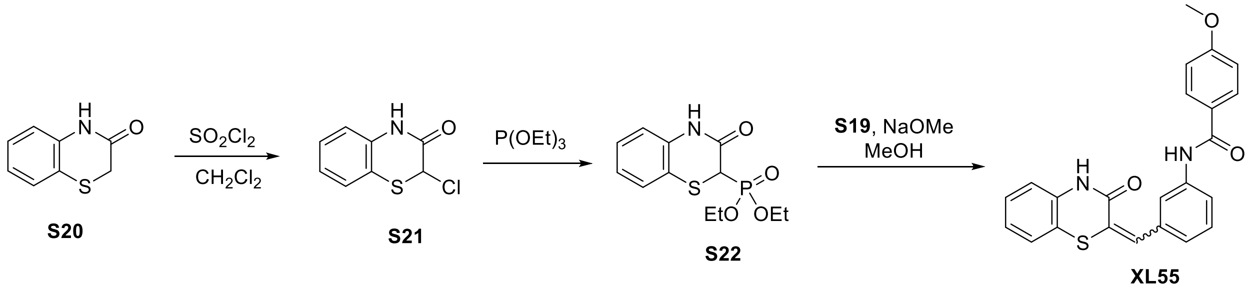

Intermediate **S22** was synthesized using the reported procedure starting from **S20**.[3] Sulfuryl chloride (162 μL, 2 mmol) was added to a stirred solution of 2,4-dihydro-1,4-benzothiazin-3-one **S20** (300 mg, 1.82 mmol) in CH_2Cl_2 (10 mL) at room temperature. After the disappearance of starting material (monitored by LC-MS), solvent was removed and the product was recrystallized in CH_2Cl_2 to provide **S21** (335 mg, 92%). ^{1}H NMR (400 MHz, dmso) δ 10.87 (s, 1H), 7.33 (dd, *J* = 7.7, 1.4 Hz, 1H), 7.19 (ddd, *J* = 8.0, 7.3, 1.4 Hz, 1H), 7.05 – 6.95 (m, 2H), 5.11 (d, *J* = 1.0 Hz, 1H). ^{13}C NMR (101 MHz, dmso) δ 161.24, 136.83, 128.88, 127.48, 123.63, 117.52, 115.87, 79.30. HRMS (m/z): $[M-Cl]^+$ calcd. for C_8H_6NOS, 164.0170; found, 164.0166.

In a sealed tube, 2-chloro-4H-1,4-benzothiazin-3-one (255 mg, 1.28 mmol) and triethyl phosphite (1.1 mL, 6.39 mmol) were heated at 120 °C for 18 hours. The reaction was concentrated and dried to afford yellow solid **S22** which was used directly for the next step. ^{1}H NMR (400 MHz, dmso) δ 10.78 (s, 1H), 7.30 (dd, *J* = 7.8, 1.4 Hz, 1H), 7.14 (td, *J* = 7.7, 1.5 Hz, 1H), 7.01 – 6.83 (m, 2H), 4.38 (d, *J* = 20.8 Hz, 1H), 4.02 – 3.67 (m, 4H), 1.12 (t, *J* = 7.0 Hz, 3H), 0.93 (t, *J* = 7.0 Hz, 3H). ^{13}C NMR (101 MHz, dmso) δ 161.65 (d), 137.50, 127.42, 127.41, 123.55, 117.12, 117.09, 63.28 (d), 63.04 (d), 37.60 (d), 16.46 (d),

16.25 (d). ^{31}P NMR (162 MHz, DMSO) δ 17.76. HRMS (m/z): $[M-Cl]^+$ calcd. for $C_{12}H_{17}NO_4PS$, 302.0616; found, 302.0617.

Aldehyde **S19** (20 mg, 0.08 mmol) and diethyl (3-oxo-3,4-dihydro-2H-1,4-benzothiazin-2-yl)phosphonate **S22** (24 mg, 0.08 mmol) were dissolved in MeOH (5 mL), and then 3.6 M NaOMe (145 µL, 0.52 mmol) in MeOH was added at room temperature. The formation of yellow precipitate was observed immediately after the addition of NaOMe. The reaction was stirred for about 2 hours and then yellow precipitate was collected. The collected precipitate was washed with cold MeOH (3 x 5 mL) and dried to afford pure **XL55** (31 mg, 97%). ^{1}H NMR (400 MHz, dmso) δ 11.02 (s, 1H), 10.24 (s, 1H), 8.08 (d, *J* = 2.2 Hz, 1H), 7.98 (d, *J* = 8.6 Hz, 2H), 7.83 – 7.78 (m, 1H), 7.75 (s, 1H), 7.45 (t, *J* = 7.9 Hz, 1H), 7.39 – 7.26 (m, 2H), 7.22 – 7.12 (m, 1H), 7.10 – 7.04 (m, 3H), 7.02 – 6.98 (m, 1H), 3.83 (s, 3H). ^{13}C NMR (101 MHz, dmso) δ 165.56, 162.45, 158.89, 140.08, 135.04, 134.62, 131.06, 130.16, 129.34, 127.43, 127.27, 125.60, 125.50, 123.68, 121.63, 121.26, 121.12, 117.23, 115.48, 114.09, 55.91. HRMS (m/z): $[M+H]^+$ calcd. for $C_{23}H_{19}N_2O_3S$, 403.1116; found, 403.1106.

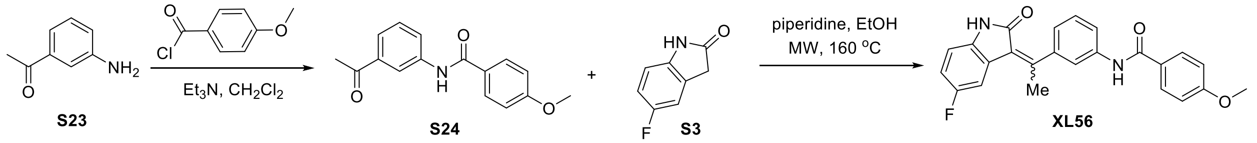

To a stirred solution of 3'-aminoacetophenone **S23** (500 mg, 3.7 mmol) in CH_2Cl_2 (10 mL), 4-methoxybenzoyl chloride (530 µL, 3.9 mmol), and Et_3N (800 µL, 5.5 mmol) were added at 0 °C. The reaction was warmed to room temperature and then stirred for 15 hours. Water (10 mL) and CH_2Cl_2 (20 mL) were added to the reaction mixture. Aqueous layer

was removed and the organic layer was washed with aqueous $NaHCO_3$ (20 mL) and aqueous NaCl (20 mL). The organic phase was dried (Na_2SO_4) and concentrated to afford N-(3-acetylphenyl)-4-methoxybenzamide **S24**, which was used for the next step without further purification. ^{1}H NMR (400 MHz, cdcl$_3$) δ 8.26 (s, 1H), 8.16 (t, *J* = 1.9 Hz, 1H), 8.05 (ddd, *J* = 8.1, 2.3, 1.0 Hz, 1H), 7.92 – 7.83 (m, 2H), 7.73 – 7.66 (m, 1H), 7.44 (t, *J* = 7.9 Hz, 1H), 6.99 – 6.89 (m, 2H), 3.86 (s, 3H), 2.59 (s, 3H). 13C NMR (101 MHz, cdcl3) δ 198.10, 165.53, 162.66, 138.81, 137.73, 129.34, 129.07, 126.67, 124.88, 124.12, 119.70, 113.98, 55.47, 26.68. HRMS (m/z): $[M+H]^+$ calcd. for $C_{16}H_{16}NO_3$, 270.1130; found, 270.1131.

In a thick-walled microwave vial, N-(3-acetylphenyl)-4-methoxybenzamide **S24** (100 mg, 0.37 mmol) and 5-fluoroindolin-2-one **S3** (280 mg, 1.85 mmol) were dissolved in EtOH (10 mL), and then piperidine (73 µL, 0.73 mmol) was added. The vial was sealed and irradiated in a microwave at 150 ^oC for 4 hours (~ 80% conversion by LC-MS). The cooled reaction mixture was dissolved in CH_2Cl_2 (30 mL) and washed with aqueous NH_4Cl (10 mL) followed by aqueous NaCl (20 mL). The organic layer was dried over anhydrous Na_2SO_4, concentrated, and purified by ISCO combi flash silica gel column (hexanes/EtOAc) to provide **XL56** (48 mg, 32%). ^{1}H NMR (400 MHz, cdcl$_3$) δ 8.11 (s, 1H), 8.00 (s, 1H), 7.91 – 7.82 (m, 2H), 7.73 (dt, *J* = 8.2, 1.5 Hz, 1H), 7.64 (t, *J* = 1.9 Hz, 1H), 7.48 (t, *J* = 7.9 Hz, 1H), 7.03 (dt, *J* = 7.6, 1.3 Hz, 1H), 7.00 – 6.93 (m, 2H), 6.80 – 6.64 (m, 2H), 5.93 (dd, *J* = 9.7, 2.5 Hz, 1H), 3.87 (s, 3H), 2.79 (s, 3H). ^{13}C NMR (101 MHz, cdcl$_3$) δ 169.34, 165.20, 162.66, 156.58, 143.12, 139.24, 135.28, 130.18, 128.93, 126.78, 121.94, 120.03, 117.49, 114.68, 114.44, 114.05, 111.02, 110.75, 109.39, 109.31, 55.48,

22.86. ^{19}F NMR (376 MHz, cdcl$_3$) δ -121.28 (td). HRMS (m/z): $[M+H]^+$ calcd. for $C_{24}H_{20}FN_2O_3$, 403.1458; found, 403.1445.

Reference:

1. Lu, X., Sabbasani, V.R., Osei-Amponsa, V., Evans, C.N., King, J.C., Tarasov, S.G., Dyba, M., Das, S., Chan, K.C., Schwieters, C.D., et al. (2021). Structure-guided bifunctional molecules hit a DEUBAD-lacking hRpn13 species upregulated in multiple myeloma. Nat Commun 12, 7318.
2. Khairnar, P. V., Su, Y-H., Chen, Y-C., Edukondalu, A., Chen, Y-R., and Lin, W. (2020). Organophosphane-Catalyzed Direct β-Acylation of 4-Arylidene Pyrazolones and 5-Arylidene Thiazolones with Acyl Chlorides. Org. Lett. *22*, 6868–6872.
3. Worley, J. W., Ratts, K. W., and Cammack, K. L. (1975). 2-Dialkylphosphonyl- and 2-alkylidene-3,4-dihydro-3-oxo-2H-1,4-benzothiazines. J. Org. Chem. *40*, 1731–1734.

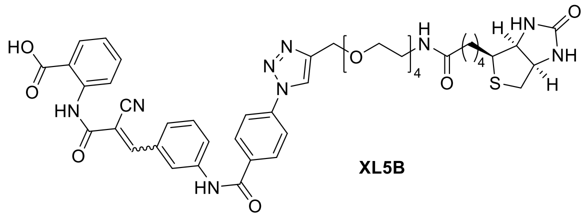

XL5B

XL5B ^{1}H NMR in DMSO-d_6

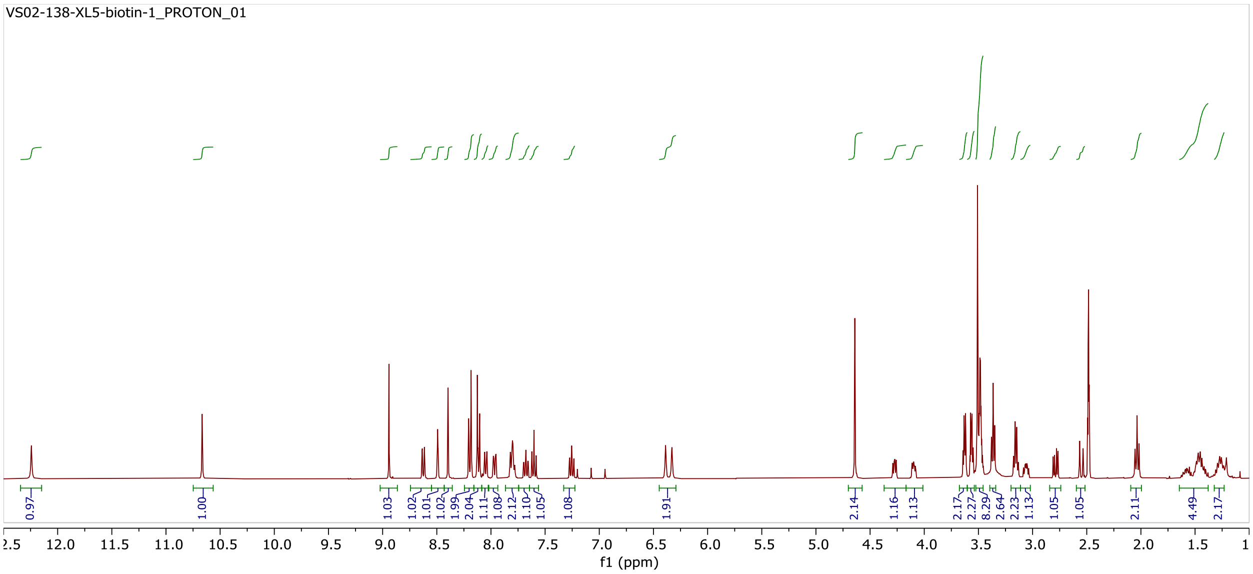

XL5B ^{13}C NMR in DMSO-d_6

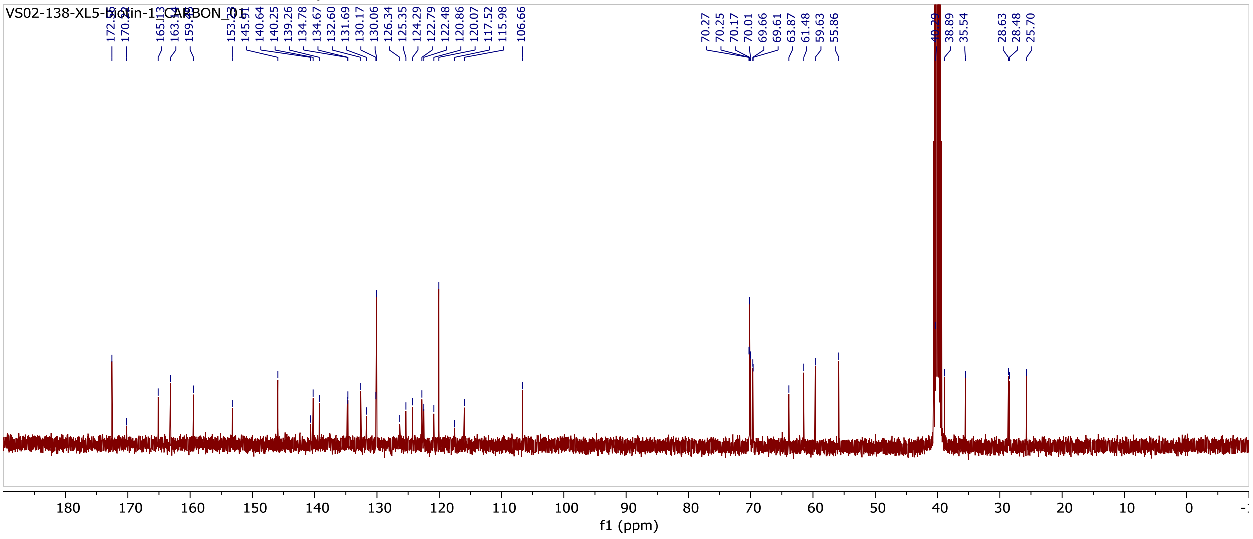

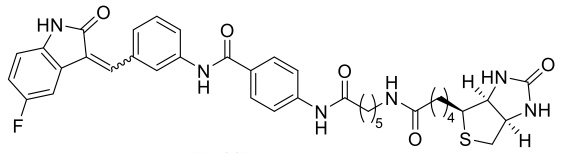

XL44B

XL44B ^{1}H NMR in DMSO-d_6

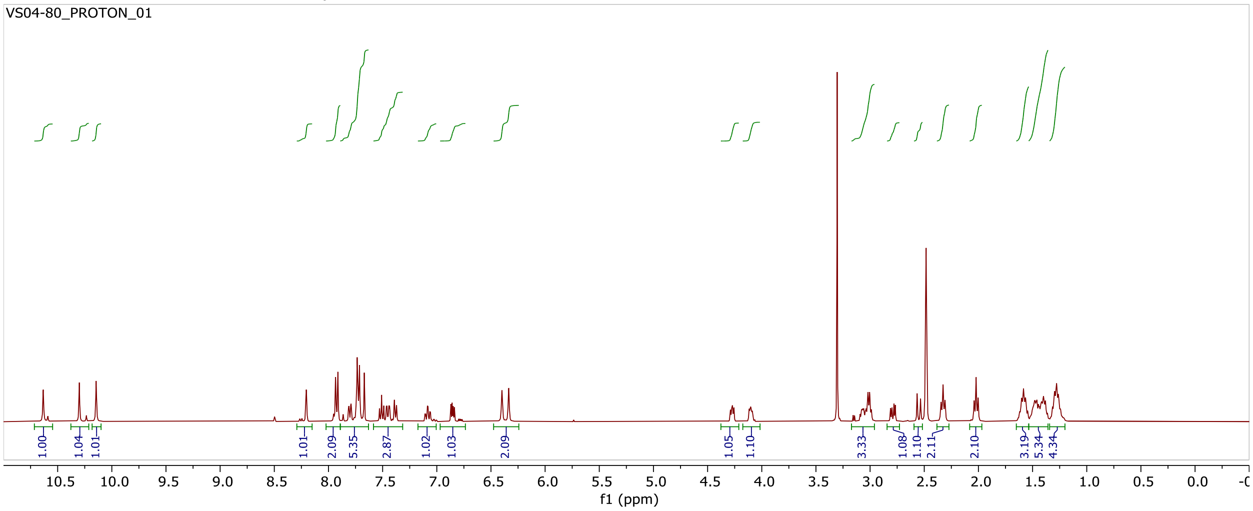

XL44B ^{13}C NMR in DMSO-d_6

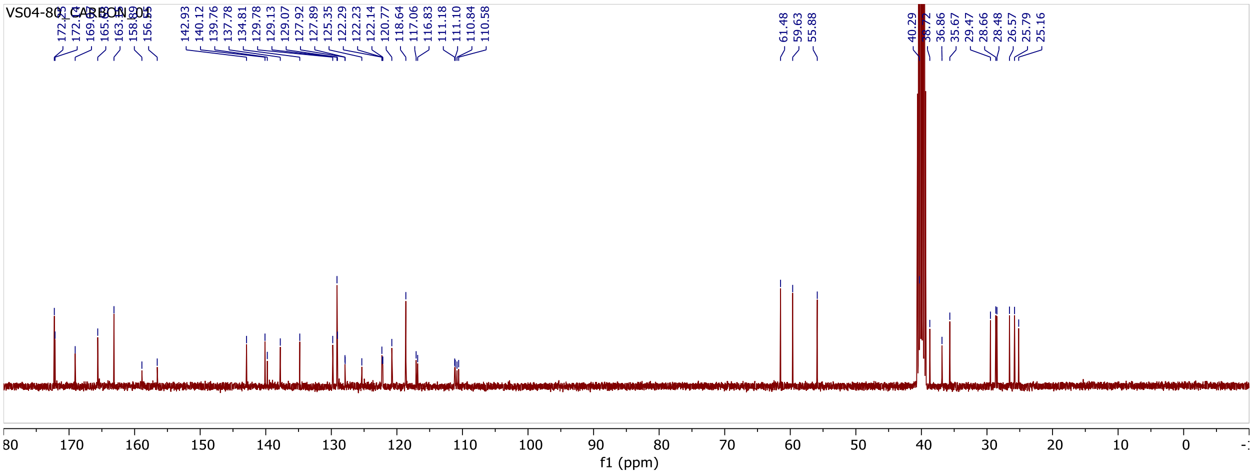

XL44B ^{19}F NMR in DMSO-d_6

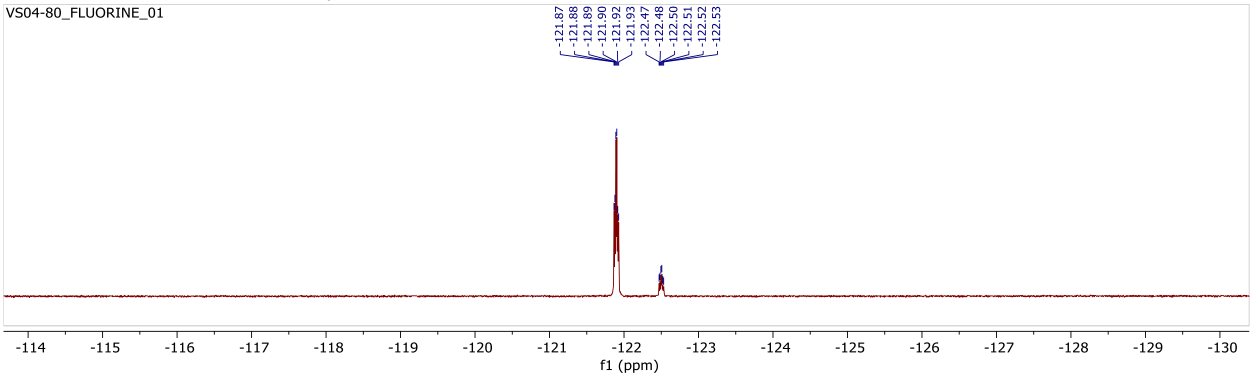

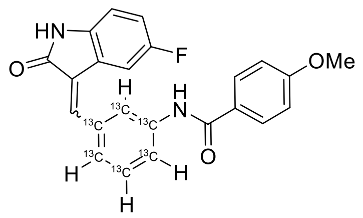

XL44-$^{13}C_6$-CB

XL44-$^{13}C_6$-CB ^{1}H NMR in DMSO-d_6

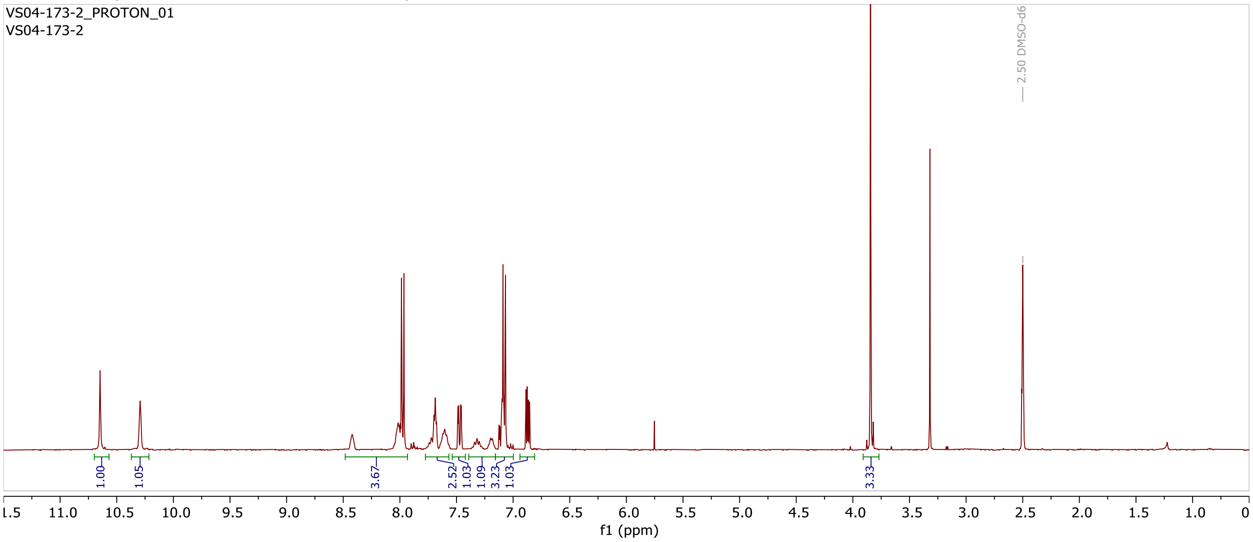

XL44-$^{13}C_6$-CB ^{1}H Bilevel Dec NMR in DMSO-d_6

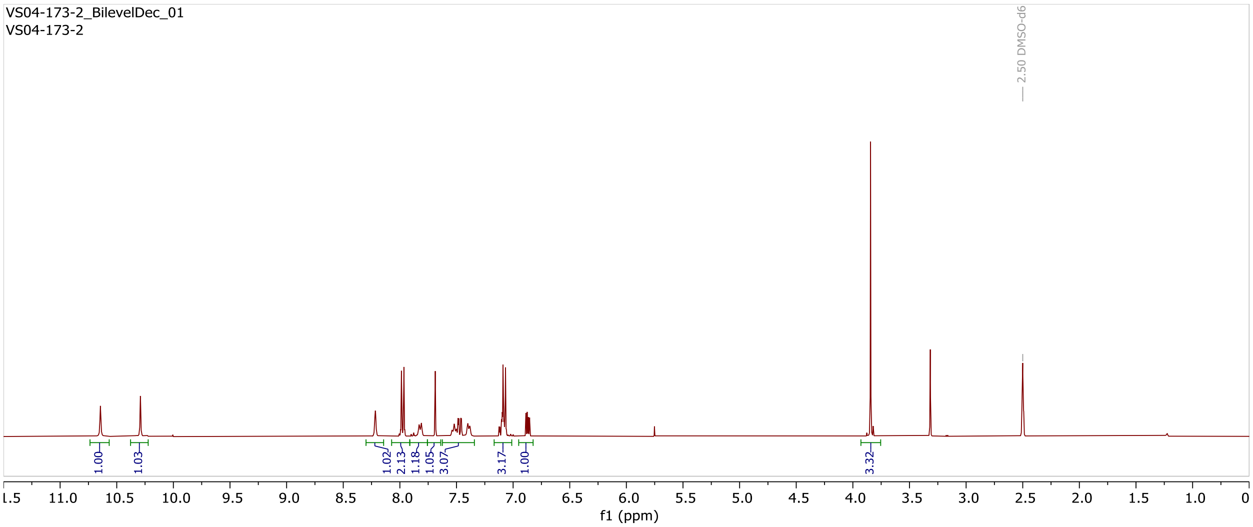

Comparison of **XL44** ^{1}H NMR and **XL44-^{13}C$_6$-CB** ^{1}H Bilevel Dec NMR in DMSO-d_6

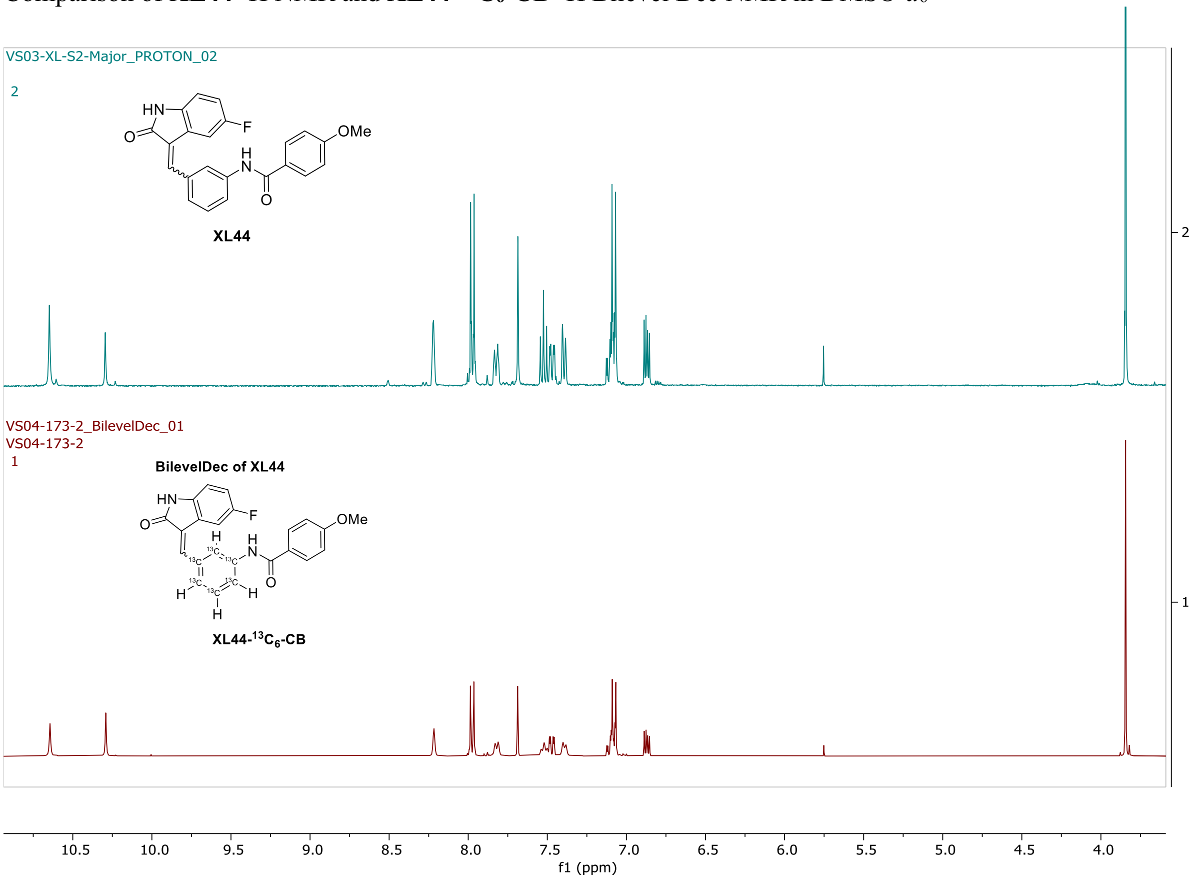

XL44-^{13}C$_6$-CB ^{13}C NMR in DMSO-d_6

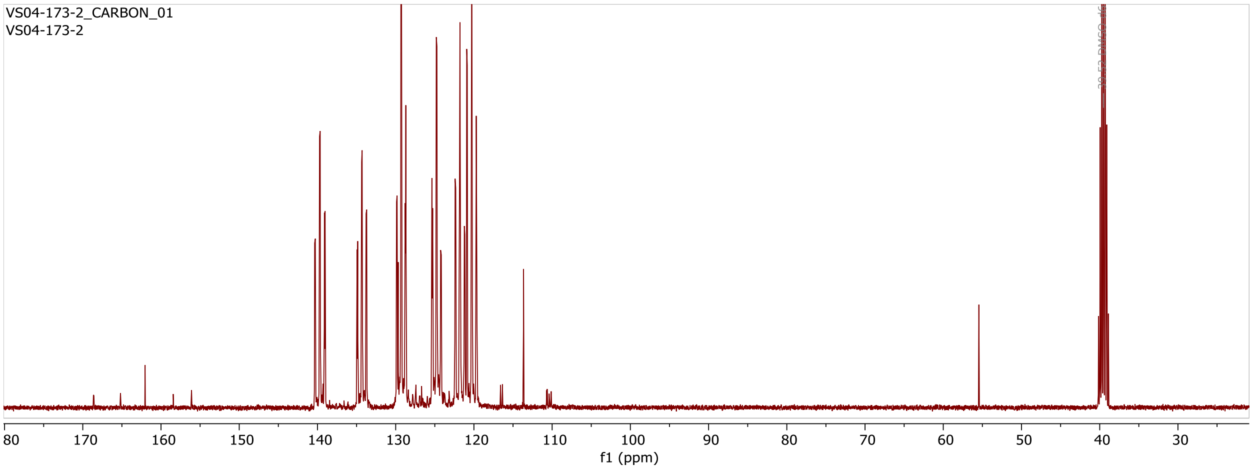

XL44-^{13}C$_6$-CB ^{19}F NMR in DMSO-d_6

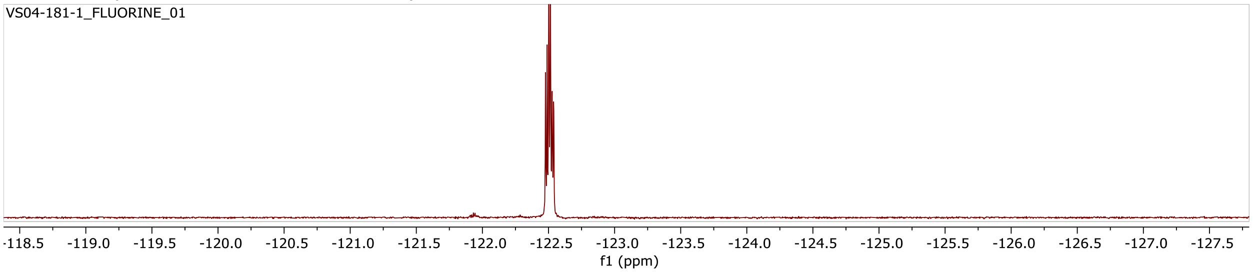

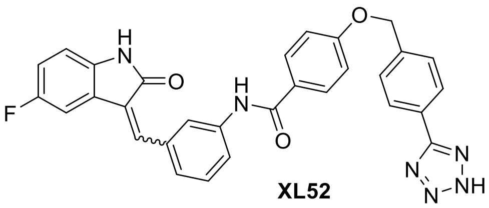

XL52

XL52 ^{1}H NMR in DMSO-d_6

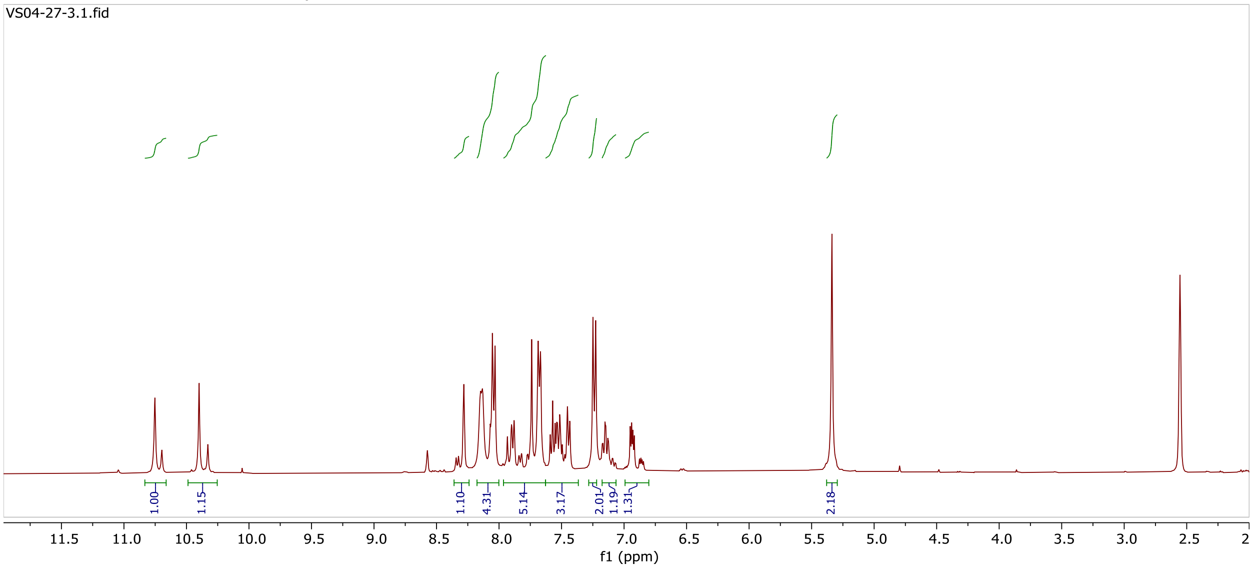

XL52 ^{13}C NMR in DMSO-d_6

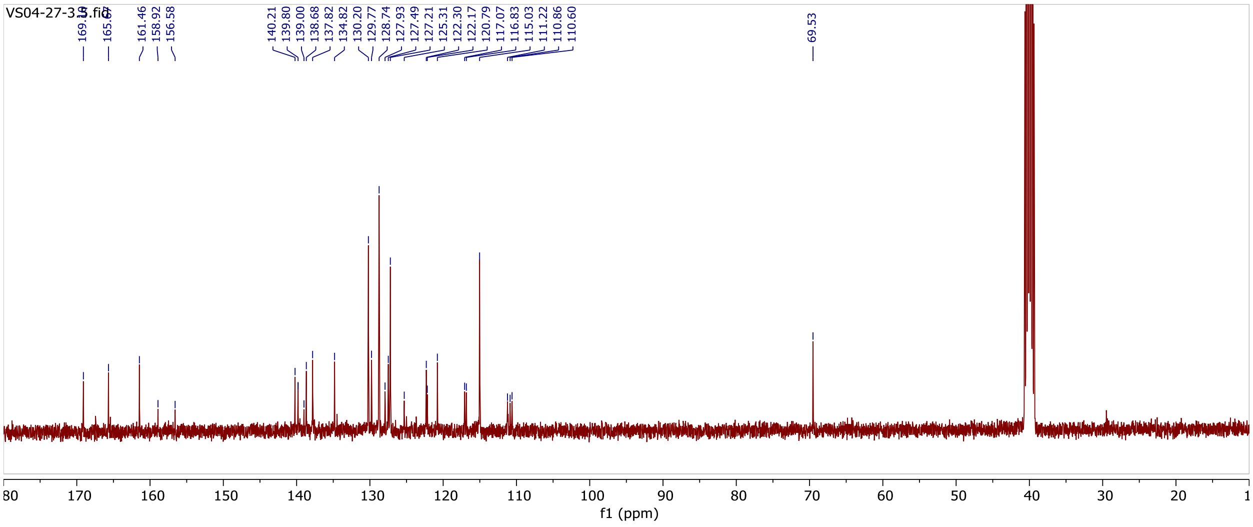

XL52 ^{19}F NMR in DMSO-d_6

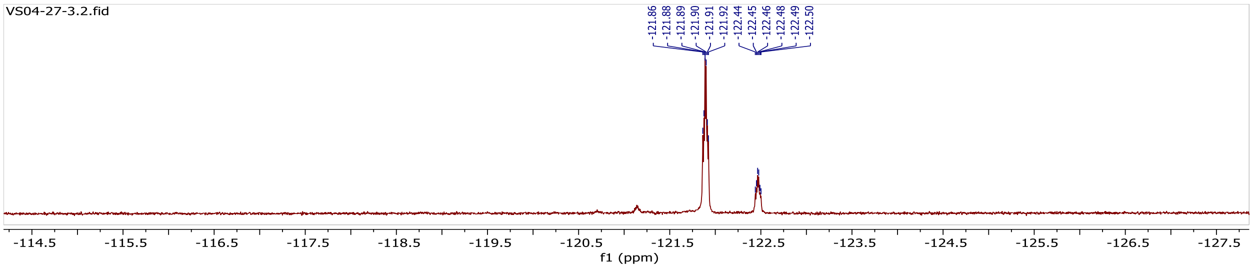

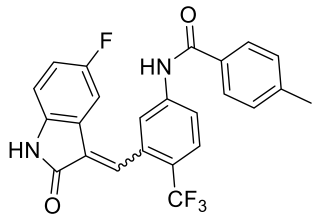

XL53

XL53 ^{1}H NMR in DMSO-d_6

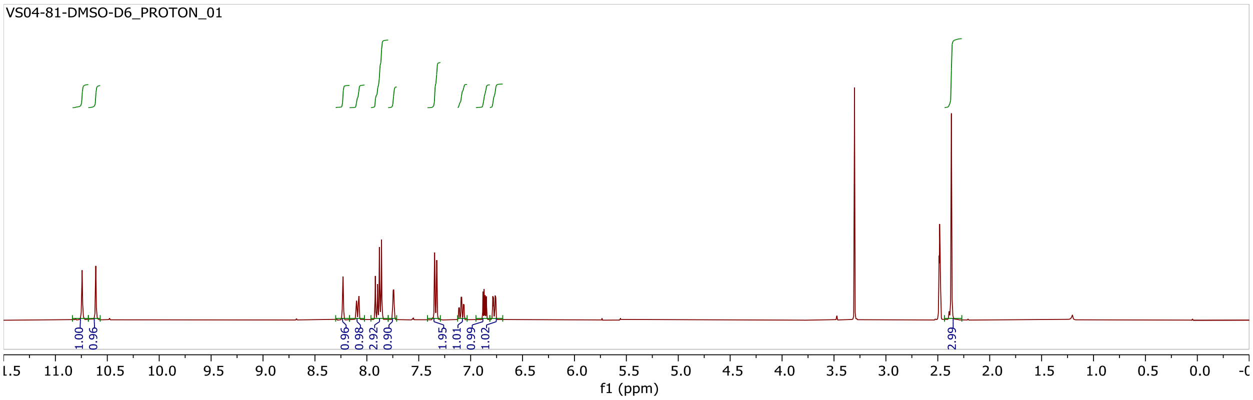

XL53 ^{13}C NMR in DMSO-d_6

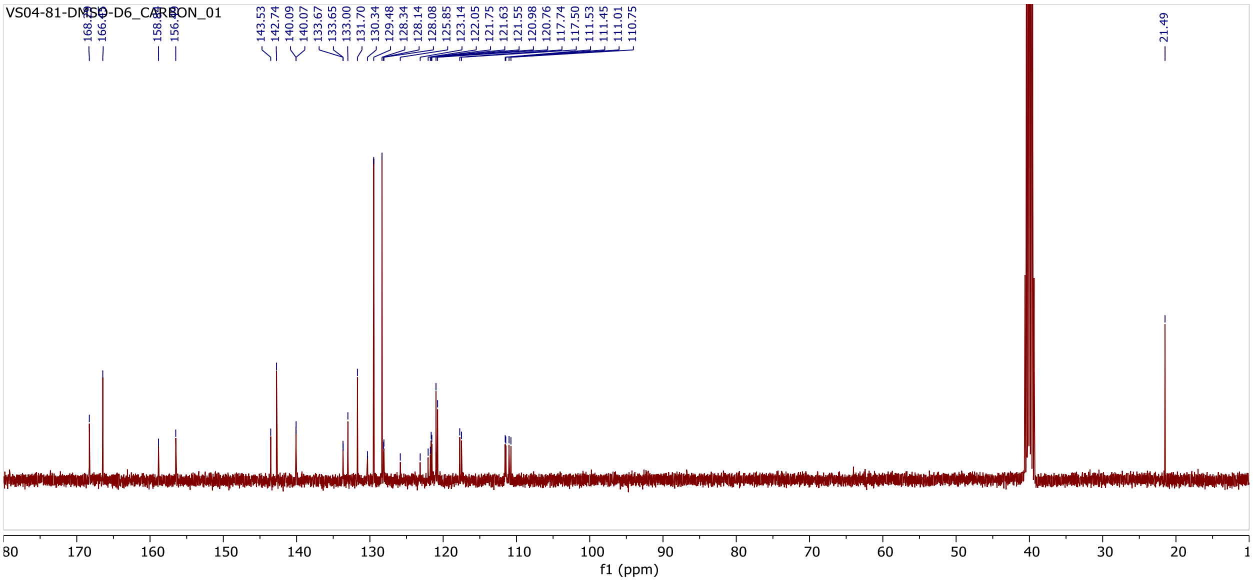

XL53 ^{19}F NMR in DMSO-d_6

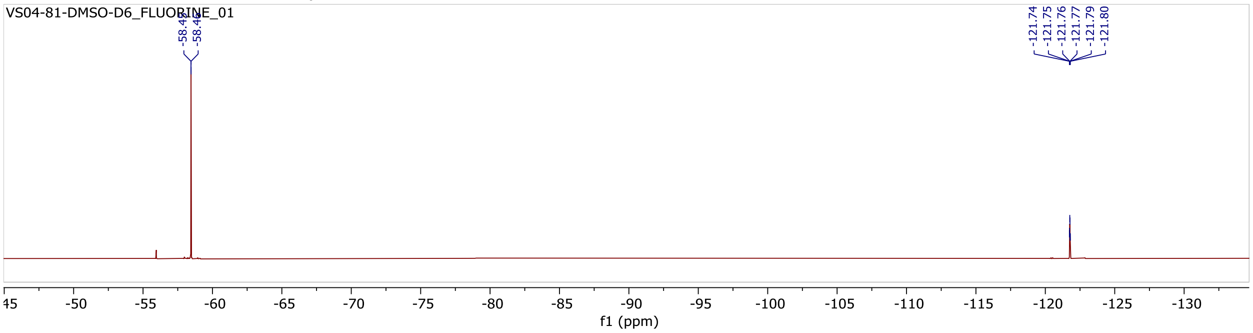

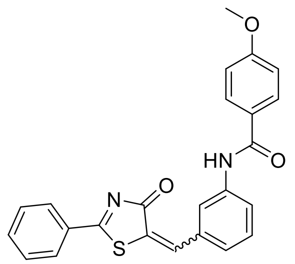

XL54

XL54 ^{1}H NMR in DMSO-d_6

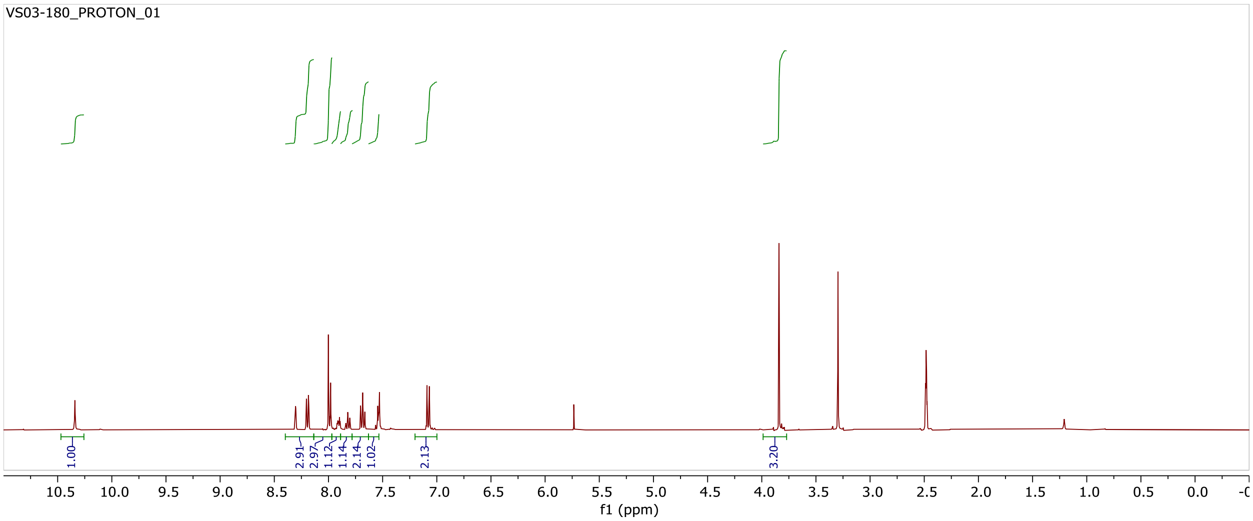

XL54 ^{13}C NMR in DMSO-d_6

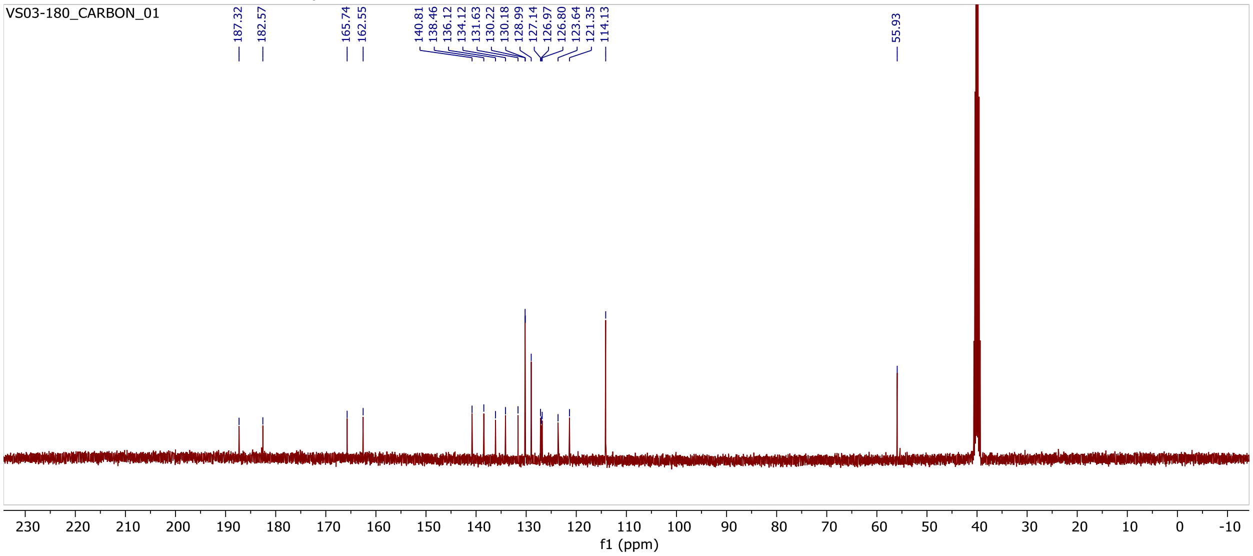

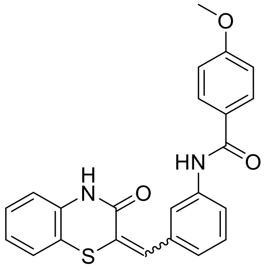

XL55

XL55 ^{1}H NMR in DMSO-d_6

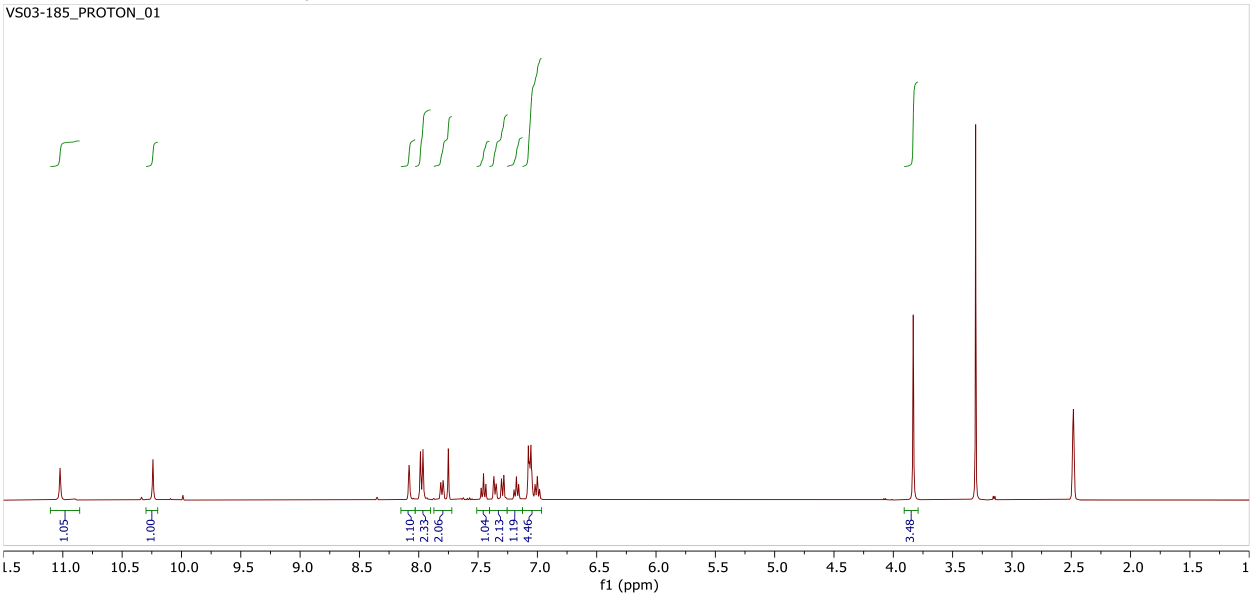

XL55 ^{13}C NMR in DMSO-d_6

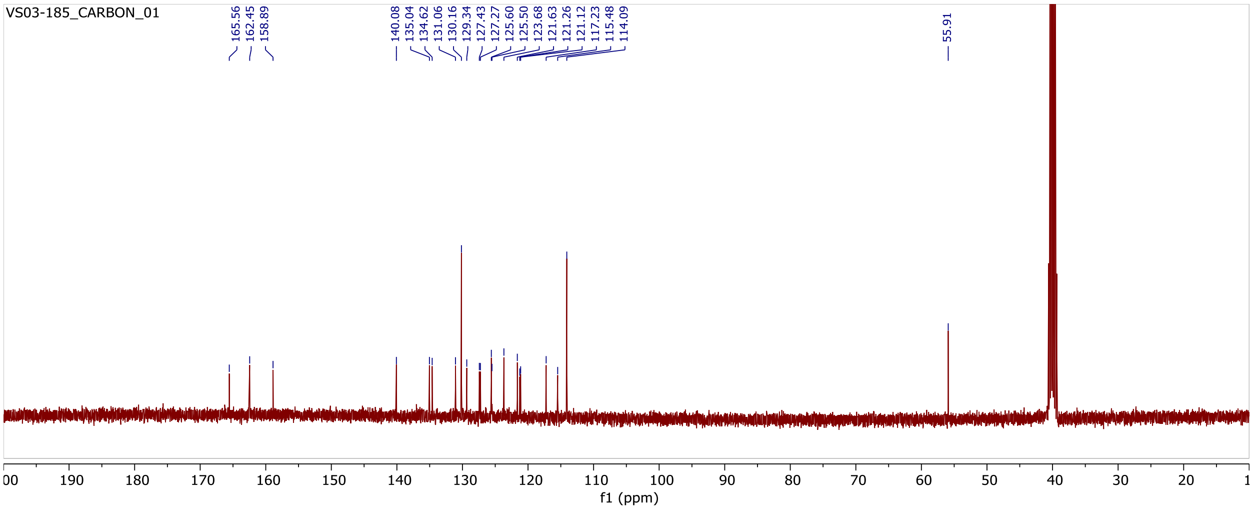

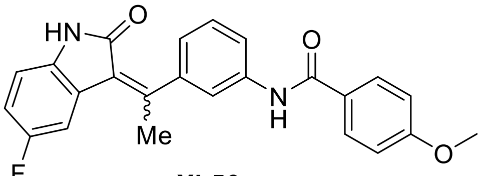

XL56

XL56 ^{1}H NMR in DMSO-d_6

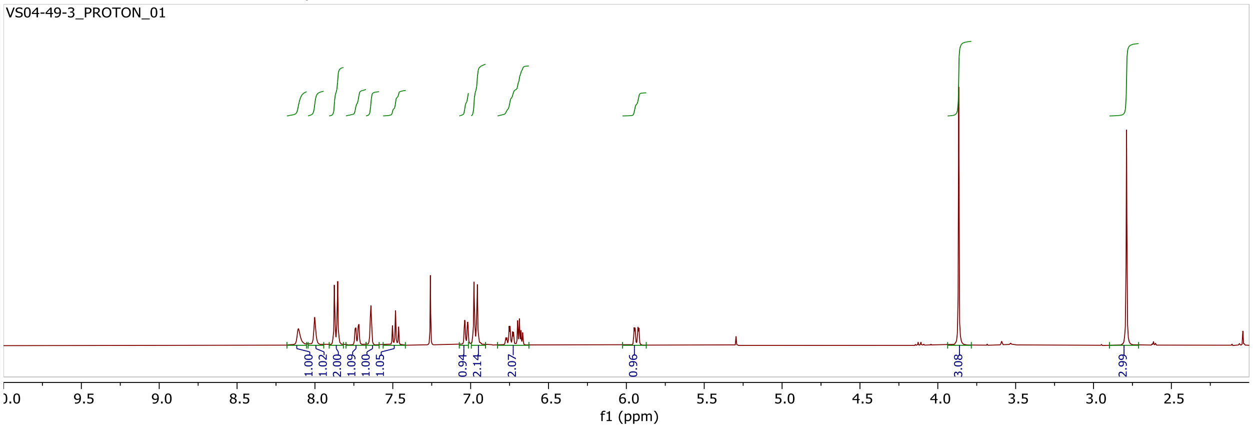

XL56 ^{13}C NMR in DMSO-d_6

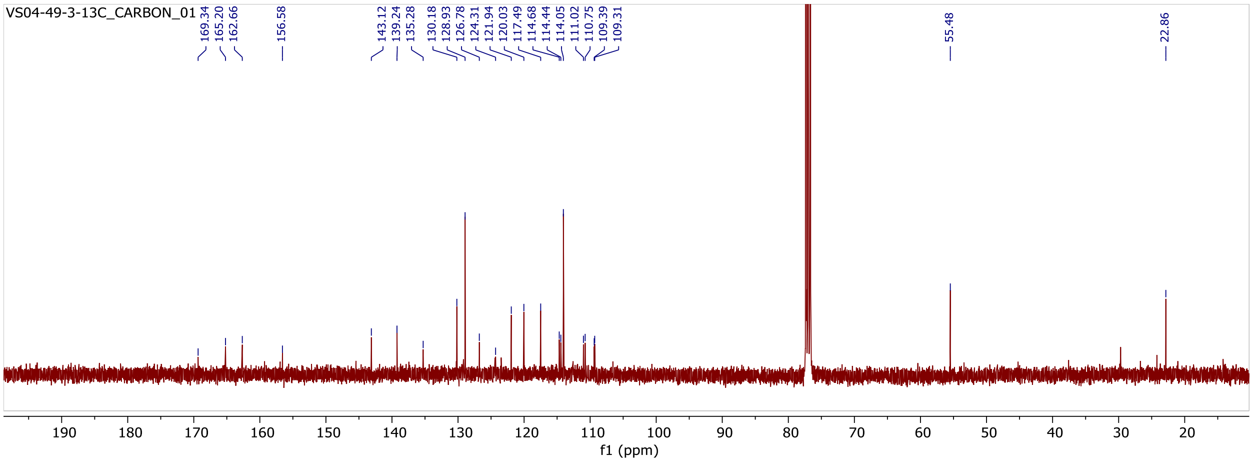

XL56 ^{19}F NMR in DMSO-d_6

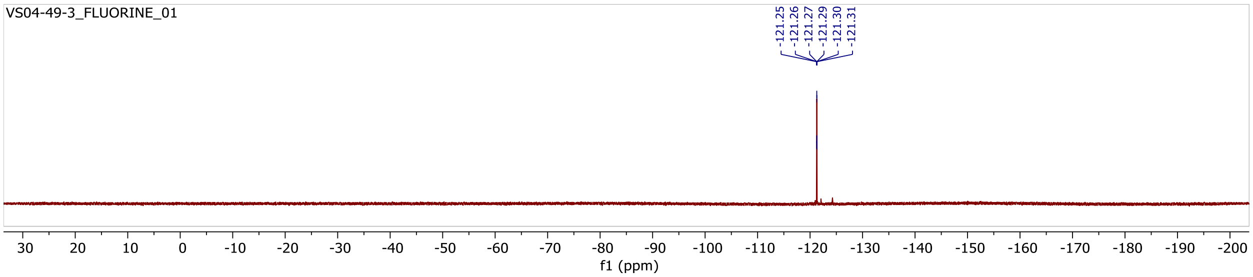

Supplement: Supplementary file 1 — Supplementary Information [file 41467_2024_46644_MOESM1_ESM.pdf]
