# Supplementary material for: A structure-based designed small molecule depletes hRpn13Pru and a select group of KEN box proteins
Source: Nat Commun. 2024 Mar 20;15:2485. doi: 10.1038/s41467-024-46644-7 (PMC10954691; doi:10.1038/s41467-024-46644-7)
Supplement: Supplementary file 2 — Reporting Summary [file 41467_2024_46644_MOESM2_ESM.pdf]

nature portfolio

Corresponding author(s): Kylie J. Walters

Last updated by author(s): Feb 12, 2024

Reporting Summary

Nature Portfolio wishes to improve the reproducibility of the work that we publish. This form provides structure for consistency and transparency in reporting. For further information on Nature Portfolio policies, see our Editorial Policies and the Editorial Policy Checklist.

Statistics

For all statistical analyses, confirm that the following items are present in the figure legend, table legend, main text, or Methods section.

n/a	Confirmed	
☐	☒	The exact sample size (*n*) for each experimental group/condition, given as a discrete number and unit of measurement
☐	☒	A statement on whether measurements were taken from distinct samples or whether the same sample was measured repeatedly
☐	☒	The statistical test(s) used AND whether they are one- or two-sided<br>*Only common tests should be described solely by name; describe more complex techniques in the Methods section.*
☒	☐	A description of all covariates tested
☒	☐	A description of any assumptions or corrections, such as tests of normality and adjustment for multiple comparisons
☐	☒	A full description of the statistical parameters including central tendency (e.g. means) or other basic estimates (e.g. regression coefficient) AND variation (e.g. standard deviation) or associated estimates of uncertainty (e.g. confidence intervals)
☐	☒	For null hypothesis testing, the test statistic (e.g. *F*, *t*, *r*) with confidence intervals, effect sizes, degrees of freedom and *P* value noted<br>*Give P values as exact values whenever suitable.*
☒	☐	For Bayesian analysis, information on the choice of priors and Markov chain Monte Carlo settings
☒	☐	For hierarchical and complex designs, identification of the appropriate level for tests and full reporting of outcomes
☒	☐	Estimates of effect sizes (e.g. Cohen's *d*, Pearson's *r*), indicating how they were calculated

Our web collection on statistics for biologists contains articles on many of the points above.

Software and code

Policy information about availability of computer code

Data collection	Schrödinger software, Bruker TopSpin 3.6.5, Prometheus NT.48 instrument, 6520 Accurate-Mass Q-TOF LC/MS system, TSQ Quantiva triple quadrupole mass spectrometer , CLARIOstar (BMG LABTECH), Omega 640 spectrophotometer, BMG Labtech, Waters Acquity UPLC system coupled with a fluorescence detector, nanoflow liquid chromatography (Thermo UltimateTM 3000RSLC nano LC system) coupled to an Orbitrap Eclipse mass spectrometer, Southeast Regional Collective Access Team (SER-CAT) using beamline ID-22 (wavelength 1.0 Å) available at the Advanced Photon Source, Argonne National Laboratory equipped with an EIGER 16M detector.
Data analysis	Schrödinger software, NMRpipe, XEASY6.4, XPLOR-NIH 3.40, PROCHECK-NMR, PyMOL, GraphPad Prism9, Mass Hunter Qualitative Analysis software (version B.07.00) , Image J, SER-CAT auto-processing software, PHENIX, and REFMAC5 (CCP4 package), Coot, Proteome Discoverer 2.4 software, percolator software, Microsoft Excel or R.

For manuscripts utilizing custom algorithms or software that are central to the research but not yet described in published literature, software must be made available to editors and reviewers. We strongly encourage code deposition in a community repository (e.g. GitHub). See the Nature Portfolio guidelines for submitting code & software for further information.

Data

Policy information about availability of data

All manuscripts must include a data availability statement. This statement should provide the following information, where applicable:

- Accession codes, unique identifiers, or web links for publicly available datasets
- A description of any restrictions on data availability
- For clinical datasets or third party data, please ensure that the statement adheres to our policy

The structural coordinate for XL44-ligated hRpn13 Pru with ubiquitin in this study has been deposited in the Protein Data Bank (PDB) under accession code 8FTQ (https://www.rcsb.org/structure/unreleased/8FTQ). The structural coordinate for XL5-ligated hRpn13 Pru mentioned in this study has been deposited in the Protein Data Bank (PDB) under accession code 7KXI. The Enamine's REAL database is publically available under the EnamineStore (https://enaminestore.com/search). TMT-MS data have been deposited at MassIVE with accession number MSV000092929 (https://massive.ucsd.edu/ProteoSAFe/dataset.jsp?task=6aa6076dfa6f4322a9539b9eb71e81ff). Source data are provided with this paper.

Research involving human participants, their data, or biological material

Policy information about studies with human participants or human data. See also policy information about sex, gender (identity/presentation), and sexual orientation and race, ethnicity and racism.

Reporting on sex and gender	N/A
Reporting on race, ethnicity, or other socially relevant groupings	N/A
Population characteristics	N/A
Recruitment	N/A
Ethics oversight	N/A

Note that full information on the approval of the study protocol must also be provided in the manuscript.

Field-specific reporting

Please select the one below that is the best fit for your research. If you are not sure, read the appropriate sections before making your selection.

☒ Life sciences ☐ Behavioural & social sciences ☐ Ecological, evolutionary & environmental sciences

For a reference copy of the document with all sections, see nature.com/documents/nr-reporting-summary-flat.pdf

Life sciences study design

All studies must disclose on these points even when the disclosure is negative.

Sample size	No statistical methods were used to predetermine sample size. Sample size for each experiment was based on the literature or previous experiences (Lu, X. et al. Structure-guided bifunctional molecules hit a DEUBAD-lacking hRpn13 species upregulated in multiple myeloma. Nat Commun 12, 7318 (2021); Lu, X. et al. Structure of the Rpn13-Rpn2 complex provides insights for Rpn13 and Uch37 as anticancer targets. Nat Commun 8:15540 (2017)) to get statistical significance or reproductivity.
Data exclusions	No data were excluded from the analysis
Replication	Biophysical experiments and vitro assay including 2D NMR, DSF, MST, LC-MS and hRpn13 degradation assay were repeated at least once. Experiments using mammalian cells were repeated at least once. All replications were consistent. Only Experiments in Fig. 6a, 6d, 6f were performed once.
Randomization	This study did not allocate experimental groups thus randomization is not required in the reported experiments.
Blinding	Virtual screening, biophysical assays, structural determination and cell -based assays are used in this study, no human, animal or behavioral experiments were performed in this study, thus blinding was not required in this study. All experiments were done with appropriate positive or negative controls as indicated.

Reporting for specific materials, systems and methods

We require information from authors about some types of materials, experimental systems and methods used in many studies. Here, indicate whether each material, system or method listed is relevant to your study. If you are not sure if a list item applies to your research, read the appropriate section before selecting a response.

Materials & experimental systems

n/a	Involved in the study
☐	☒ Antibodies
☐	☒ Eukaryotic cell lines
☒	☐ Palaeontology and archaeology
☒	☐ Animals and other organisms
☒	☐ Clinical data
☒	☐ Dual use research of concern
☒	☐ Plants

Methods

n/a	Involved in the study
☒	☐ ChIP-seq
☒	☐ Flow cytometry
☒	☐ MRI-based neuroimaging

Antibodies

Antibodies used

anti-hRpn13 (100-200) (Abcam ab157185, EPR11449(B), 1:5,000), anti-beta-actin (Cell Signaling Technology 3700s, 8H10D10, 1:6,000 or 1:10,000), anti-cleaved caspase-9 (Cell Signaling, 52873s, E5Z7N, 1:500), anti-caspase-3 (Cell Signaling, 9662s, 1:1000), anti-PCLAF (Santa Cruz, sc-390515 HRP, G-11, 1:1000 or 1:500), anti-PCLAF (Cell Signaling, 81533s, D8E2Y, 1:1000), anti-PTTG1 (Cell Signaling, 13445s, D2B6O, 1:1000), anti-RRM2 (Abcam ab57653, 1E1, 1:1,000), anti-RRM2 (Abcam ab172476, EPR11820, 1:3,000), anti-ubiquitin (P4D1) (Cell Signaling, 3936s, P4D1, 1:1000), secondary antibodies anti-mouse (Sigma-Aldrich, A9917, 1:3,000 or 1:4,000), anti-rabbit (Life Technologies, A16110, 1:5,000) antibodies.

Validation

All antibodies used in this study were described and validated in the reported papers.
Lu, X. et al. Structure-guided bifunctional molecules hit a DEUBAD-lacking hRpn13 species upregulated in multiple myeloma. Nat Commun 12, 7318 (2021).
Lu, X. et al. Structure of the Rpn13-Rpn2 complex provides insights for Rpn13 and Uch37 as anticancer targets. Nat Commun 8:15540 (2017).
Simpson, F. et al. The PCNA-associated factor KIAA0101/p15(PAF) binds the potential tumor suppressor product p33ING1b. Exp Cell Res 312, 73-85 (2006)
Marangos, P. & Carroll, J. Securin regulates entry into M-phase by modulating the stability of cyclin B. Nat Cell Biol 10, 445-51 (2008).
Xu, M. et al. Proteomic Reveals Reasons for Acquired Drug Resistance in Lung Cancer Derived Brain Metastasis Based on a Newly Established Multi-Organ Microfluidic Chip Model. Front Bioeng Biotechnol 8, 612091 (2020)
Song, C., Pan, B., Yang, X. & Tang, W. Polyphyllin VII suppresses cell proliferation, the cell cycle and cell migration in colorectal cancer. Oncol Lett 21, 25 (2021)
Liu, X. et al. Pan-cancer analyses reveal the regulation and clinical outcome association of PCLAF in human tumors. Int J Oncol 60 (2022).

Eukaryotic cell lines

Policy information about cell lines and Sex and Gender in Research

Cell line source(s)

The RPMI 8226 (ATCC® CCL-155™), HS5-BMSCs (ATCC® CRL-3611™), HCT116 WT (ATCC® CCL-247™), and MCF7 (ATCC® HTB-22™) cell lines were purchased from the American Tissue Culture Collection; OVCAR-4 (SCC258) and OVCAR-5 (SCC259) were purchased from Millipore-Sigma; Human Fibroblast 1634 (HF-1634) cells were a generous gift from Dr. Douglas Lowy, RPMI 8226 trRpn13-MM2 or HCT116 trRpn13 cells were generated and described in our previous studies. RPMI 8226 trRpn13-MM2/hRpn13 cells were generated in this study.

Authentication

None of the commercial cell lines used in this study were further authenticated. RPMI 8226 trRpn13 MM2 cell lines were validated by sequencing PCR-amplified genomic DNA and RNA PacBio sequencing in addition to Western blotting, which was described in our previous work (Lu, X. et al. Structure-guided bifunctional molecules hit a DEUBAD-lacking hRpn13 species upregulated in multiple myeloma. Nat Commun 12, 7318 (2021)). The HCT116 trRpn13 cell line was validated by sequencing PCR-amplified genomic DNA and RNA PacBio sequencing in addition to Western blotting, which was described in our previous work (Osei-Amponsa V et al. Impact of Losing hRpn13 Pru or UCHL5 on Proteasome Clearance of Ubiquitinated Proteins and RA190 Cytotoxicity. Mol Cell Biol 40:N/A (2020).).

Mycoplasma contamination

The cell lines were not tested for mycoplasma contamination.

Commonly misidentified lines
(See ICLAC register)

No cell lines in this study were commonly misidentified lines.
